# Supplementary figures and images for: The regulation landscape of MAPK signaling cascade for thwarting Bacillus thuringiensis infection in an insect host
Source: PLoS Pathog. 2021 Sep 8;17(9):e1009917. doi: 10.1371/journal.ppat.1009917 (PMC8452011; doi:10.1371/journal.ppat.1009917)

S1 Fig

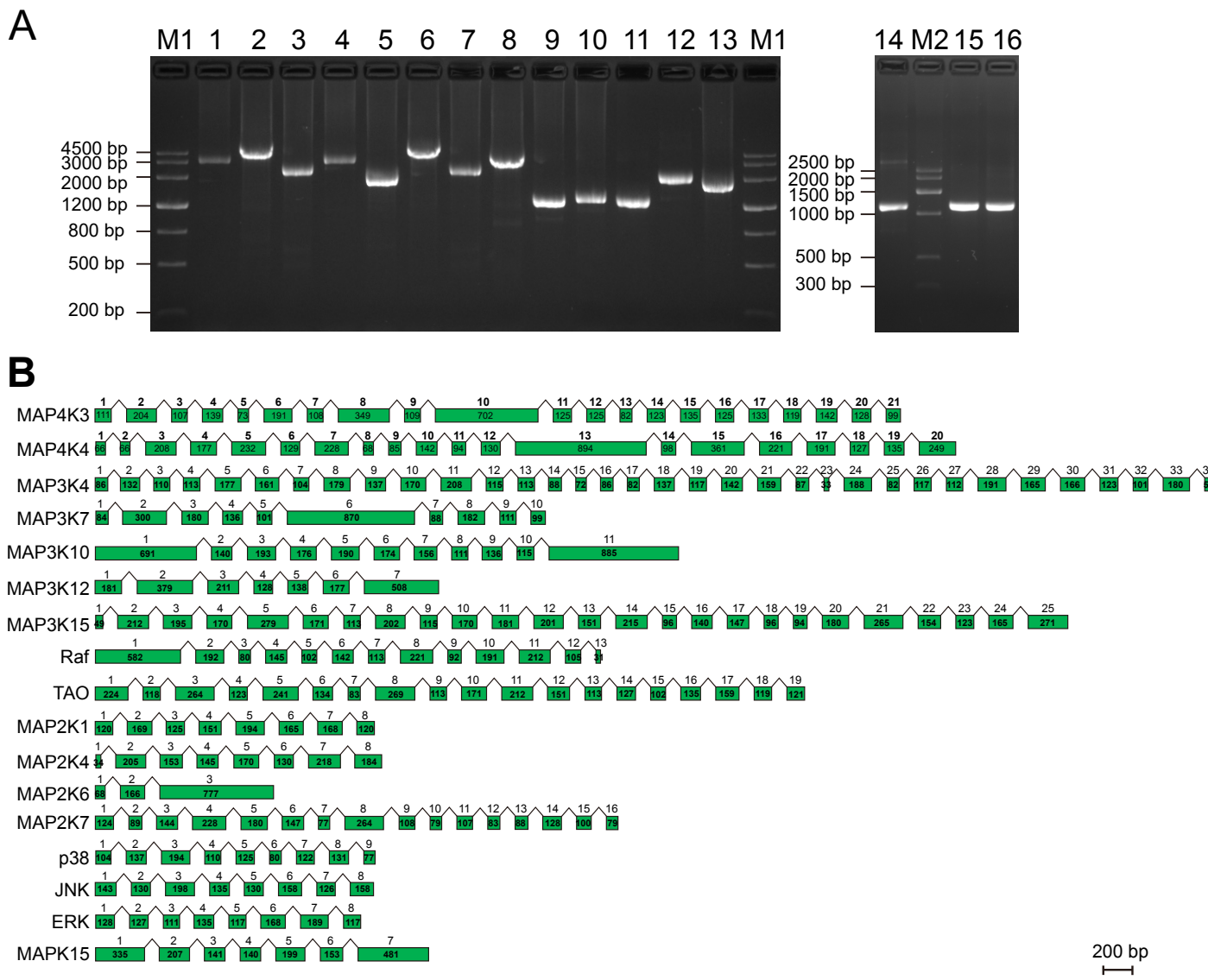

Supplement: S1 Fig — (A) Amplification of full-length cDNA of all identified MAPK cascade genes in P. xylostella. M1 and M2 represent two molecular size markers. Lanes 1 to 13 are PxMAP4K3, PxMAP3K4, PxMAP3K7, PxMAP3K10, PxMAP3K12, PxMAP3K15, PxRaf, PxTAO, PxMAP2K1, PxMAP2K4, PxMAP2K6, PxMAP2K7 and PxMAPK15 respectively. Lanes 14 to 16 are Pxp38, PxERK and PxJNK. All the PCR products were resolved by 1.5% agarose gel electrophoresis. (B) Gene structure of all identified MAPK cascade genes. The boxes represent exons and are drawn to scale. The numbers in boxes indicate the length of exons and the numbers above boxes indicate the exons order. (PDF) [file ppat.1009917.s001.pdf]

S2 Fig

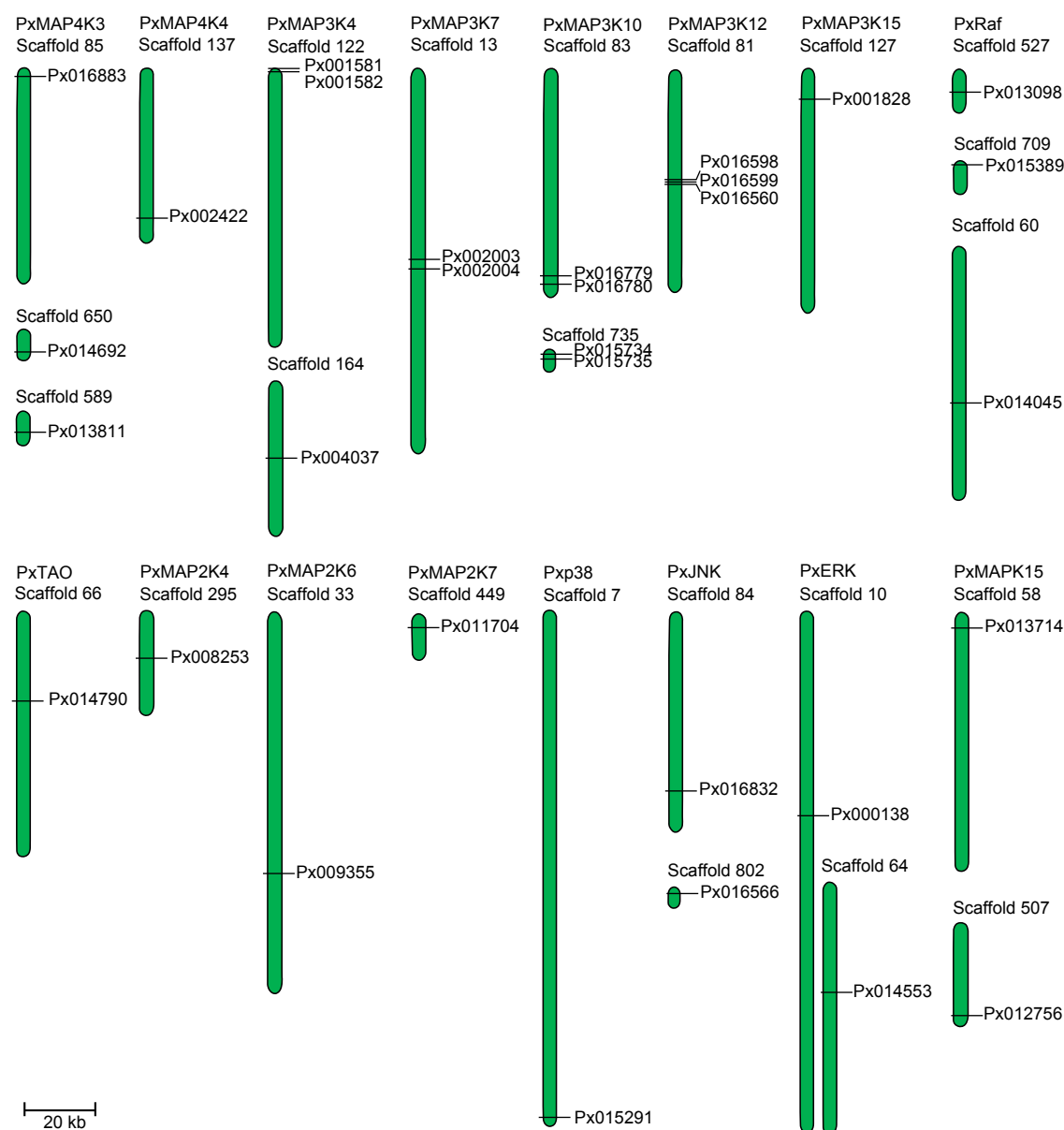

Supplement: S2 Fig — The length of scaffolds and the location of MAPK genes are drawn to scale. The sequences on the same scaffold can be assembled to the same gene. For example, Px016598, Px016599 and Px016600 can be assembled to PxMAP3K12. The gene sequence of PxMAP2K1 can be found in the P. xylostella genome, but its scaffold location information is absent. (PDF) [file ppat.1009917.s002.pdf]

S4 Fig

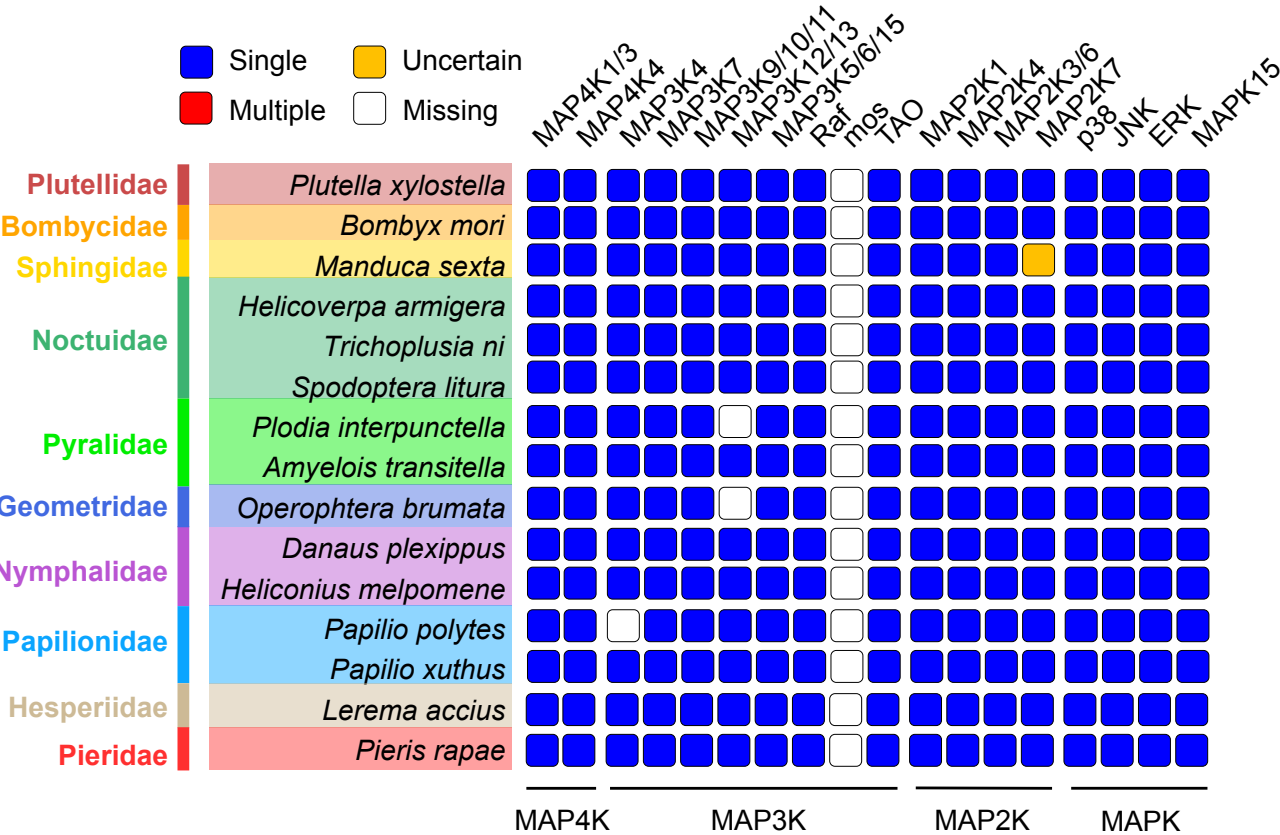

Supplement: S4 Fig — The presence or absence of the MAPKs has been assessed in the genome of 15 lepidopteran insects. The gene categories based on the classification of kinase domains are at the top. (PDF) [file ppat.1009917.s004.pdf]

S5 Fig

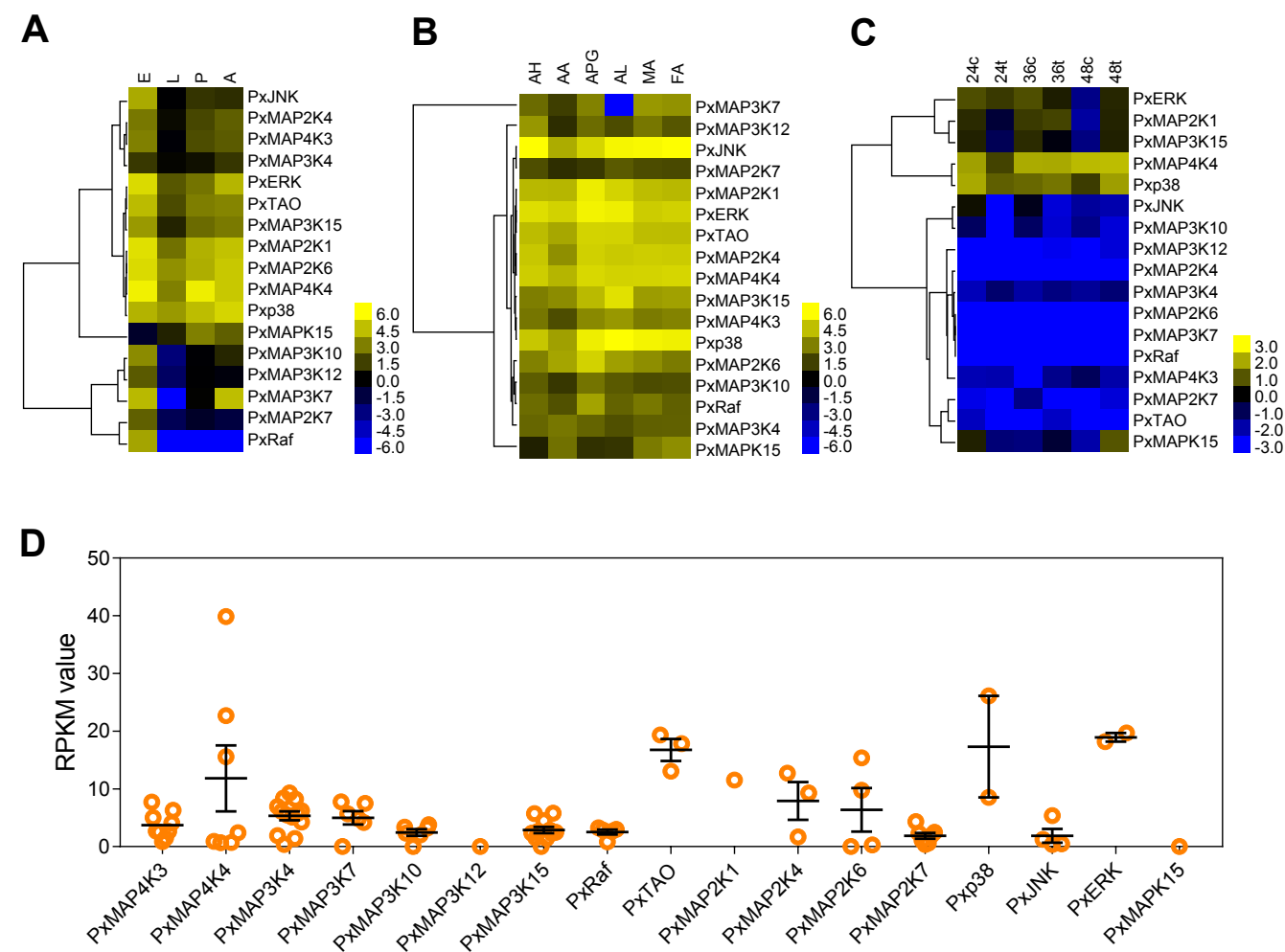

Supplement: S5 Fig — The RNA-seq data used here were downloaded from the Sequence Read Archive (SRA). (A)–(C) The log2 TPM values of genes were used to create the heatmap by Cluster 3.0. with correlation (uncentered) distance and complete linkage. Heatmaps were visualized by TreeView. (A) Expression patterns (log2 TPM values) of MAPK cascade genes in four developmental stages. E, egg (SRX056231); L, larva (SRX056232); P, pupa (SRX056233); A, adult (SRX056234). (B) Expression patterns (log2 TPM values) of MAPK cascade genes in six adult tissues. AH, adult head (SRX1984133); AA, adult abdomen (SRX1977074); APG, adult pheromone gland (SRX1984138); AL, adult leg (SRX1984145); MA, male antennae (SRX1984140); FA, female antennae (SRX1984104). (C) Expression patterns (log2 TPM values) of MAPK cascade genes in a study of fungal pathogen infection. 24c, 24 h control (SRX1165822); 24t, 24 h infection (SRX1165825); 36c, 36 h control (SRX1165823); 36t, 36 h infection (SRX1165826); 48c, 48 h control (SRX1165824); 48t, 48 h infection (SRX1165827). (D) The absolute expression levels of MAPK cascade genes in midgut tissues of third-instar DBM1Ac-S larvae as determined by the RPKM values of our previous transcriptome and RNA-seq data. The unigenes of MAPK cascade genes were identified by searching against the midgut transcriptome with the full-length cDNA sequence as queries. The RPKM values of these unigenes derived from the RNA-seq libraries were for gene expression analysis. (PDF) [file ppat.1009917.s005.pdf]

S6 Fig

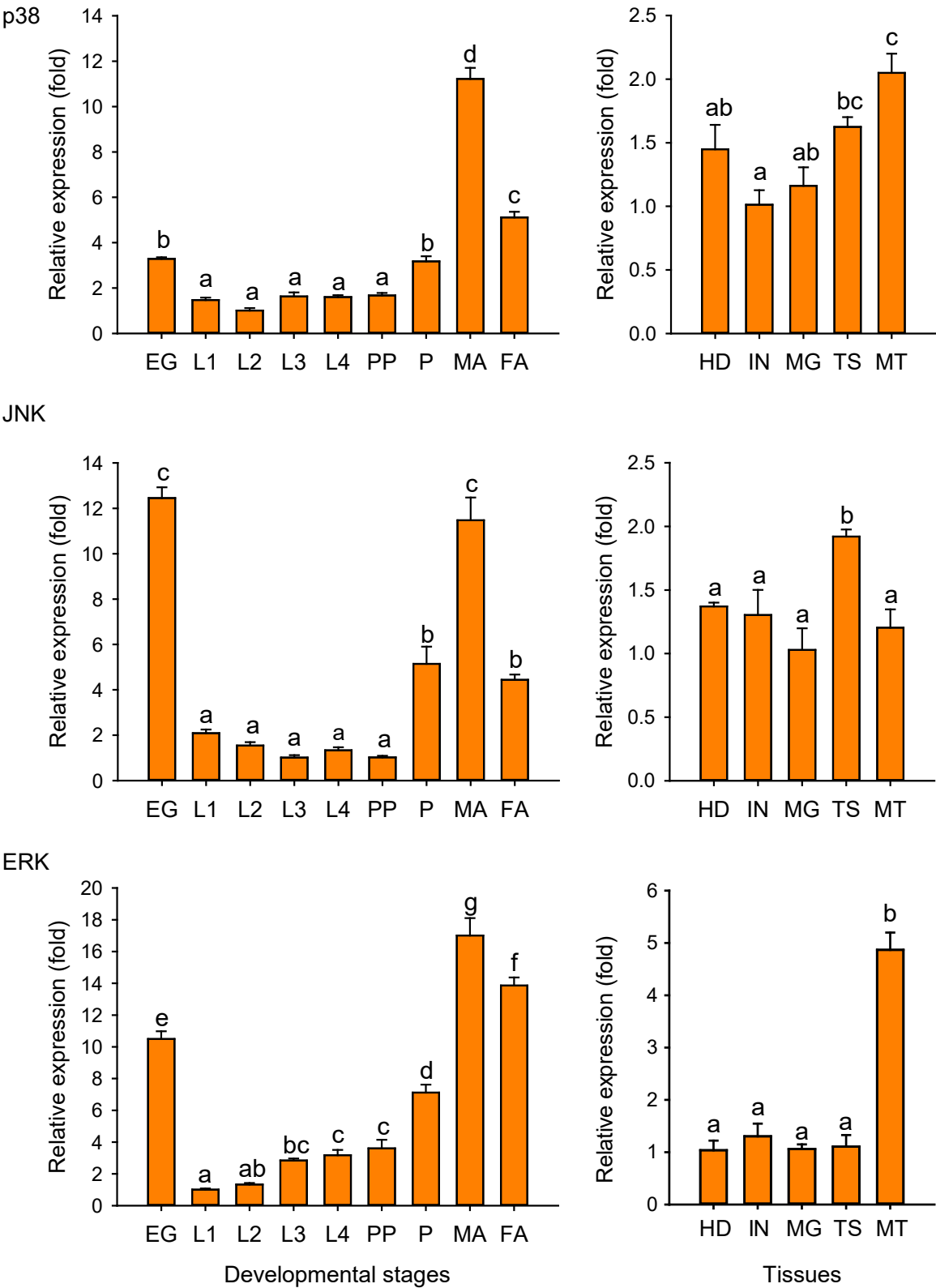

Supplement: S6 Fig — Data in the figures are means and stand errors from three biological replicates. Different letters indicate significant differences between different treatments (P < 0.05; Duncan’s test; n = 3). Developmental stages: EG, egg; L1, first-instar larvae; L2, second-instar larvae; L3, third-instar larvae; L4, fourth-instar larvae; PP, pre-pupae; P, pupae; MA, male adults; FA, female adults. Tissues: HD, head; IN, integument; MG, midgut; TS, testis; MT, Malpighian tubules. (PDF) [file ppat.1009917.s006.pdf]

S7 Fig

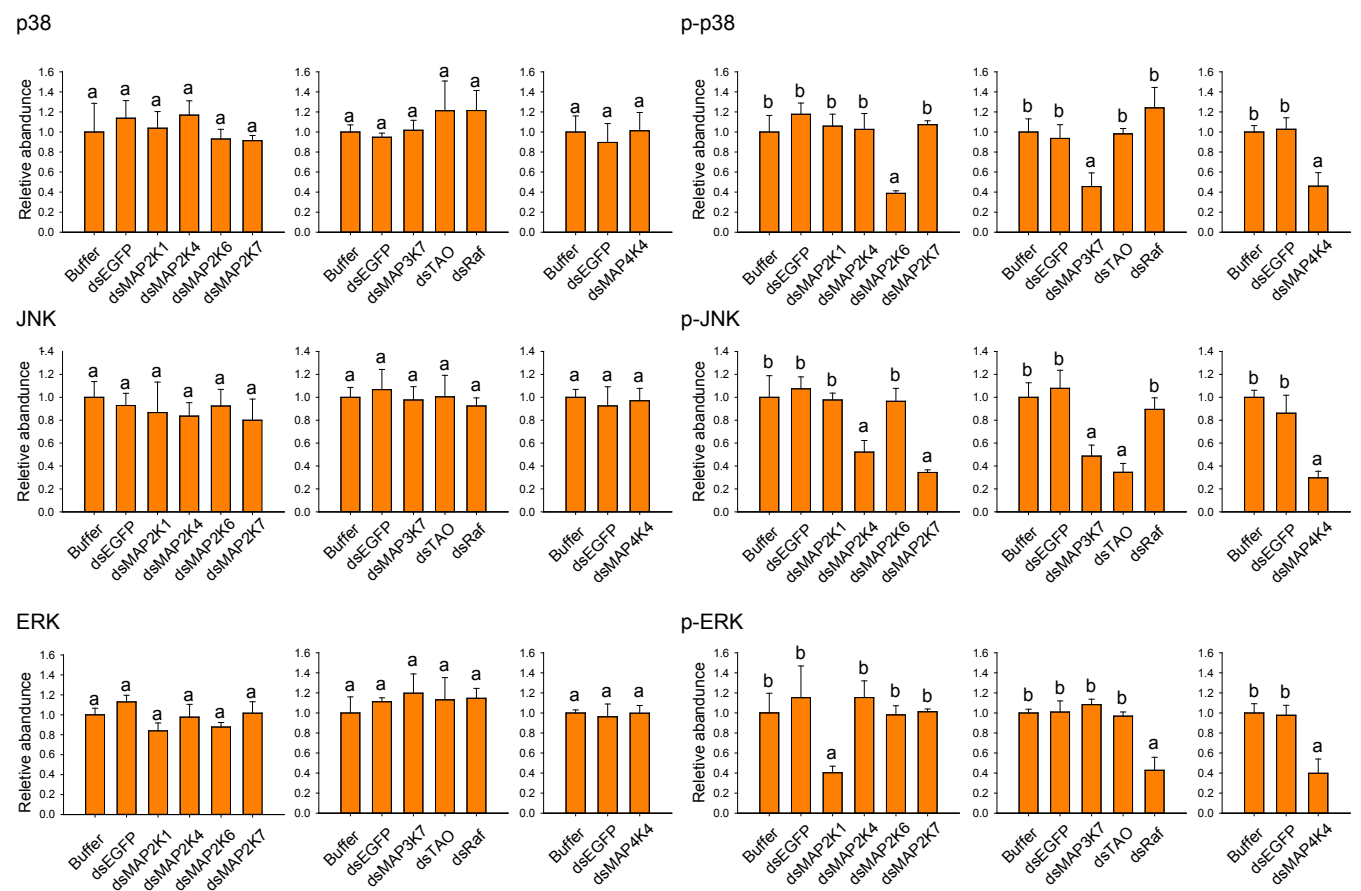

Supplement: S7 Fig — Western blot assays were analyzed with ImageJ 1.51. Data in figures show means and standard errors from three biological replicates. Different letters indicate significant differences between different treatments (P < 0.05; Duncan’s test; n = 3). (PDF) [file ppat.1009917.s007.pdf]

S8 Fig

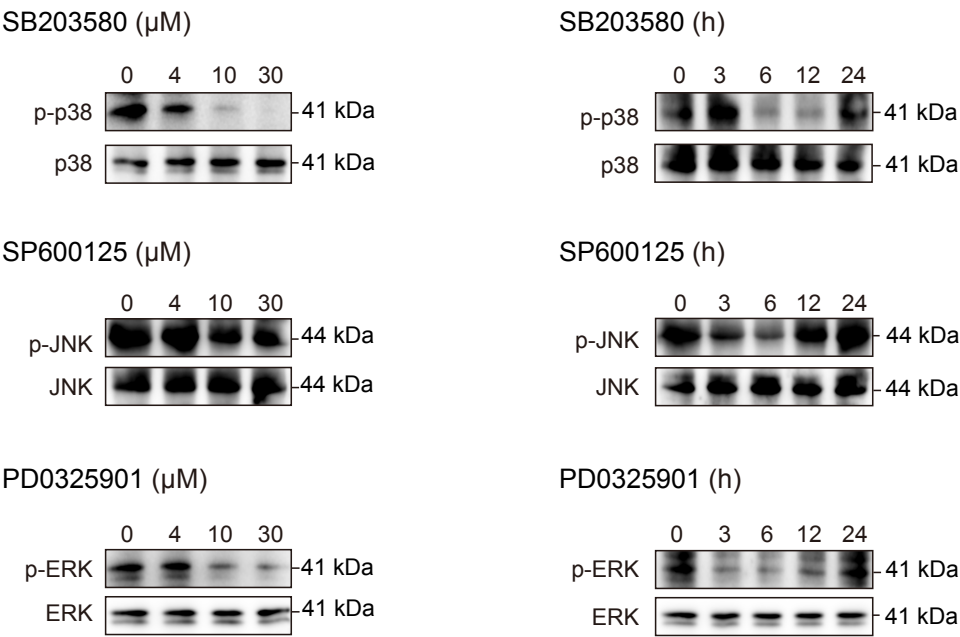

Supplement: S8 Fig — The SB203580, SP600125 and PD0325901 are the specific inhibitors of p38, JNK and ERK, respectively. To determine the appropriate concentrations of each inhibitor, the third-instar NIL-R larvae were treated with different concentrations as follows: 0, 4, 10, 30 μM for 6 h. Based on these results, the proper concentrations of inhibitors selected to be used in the subsequent assays was 30 μM for the three SB203580, SP600125 and PD0325901 inhibitors. To determine the appropriate detection time, the third-instar larvae were treated with 30 μM of each inhibitor for 0, 3, 6, 12, 24 h. All inhibitors can significantly reduce the phosphorylation level of these kinases after 6 h of treatment, which was selected as the appropriate detection time. (PDF) [file ppat.1009917.s008.pdf]

S9 Fig

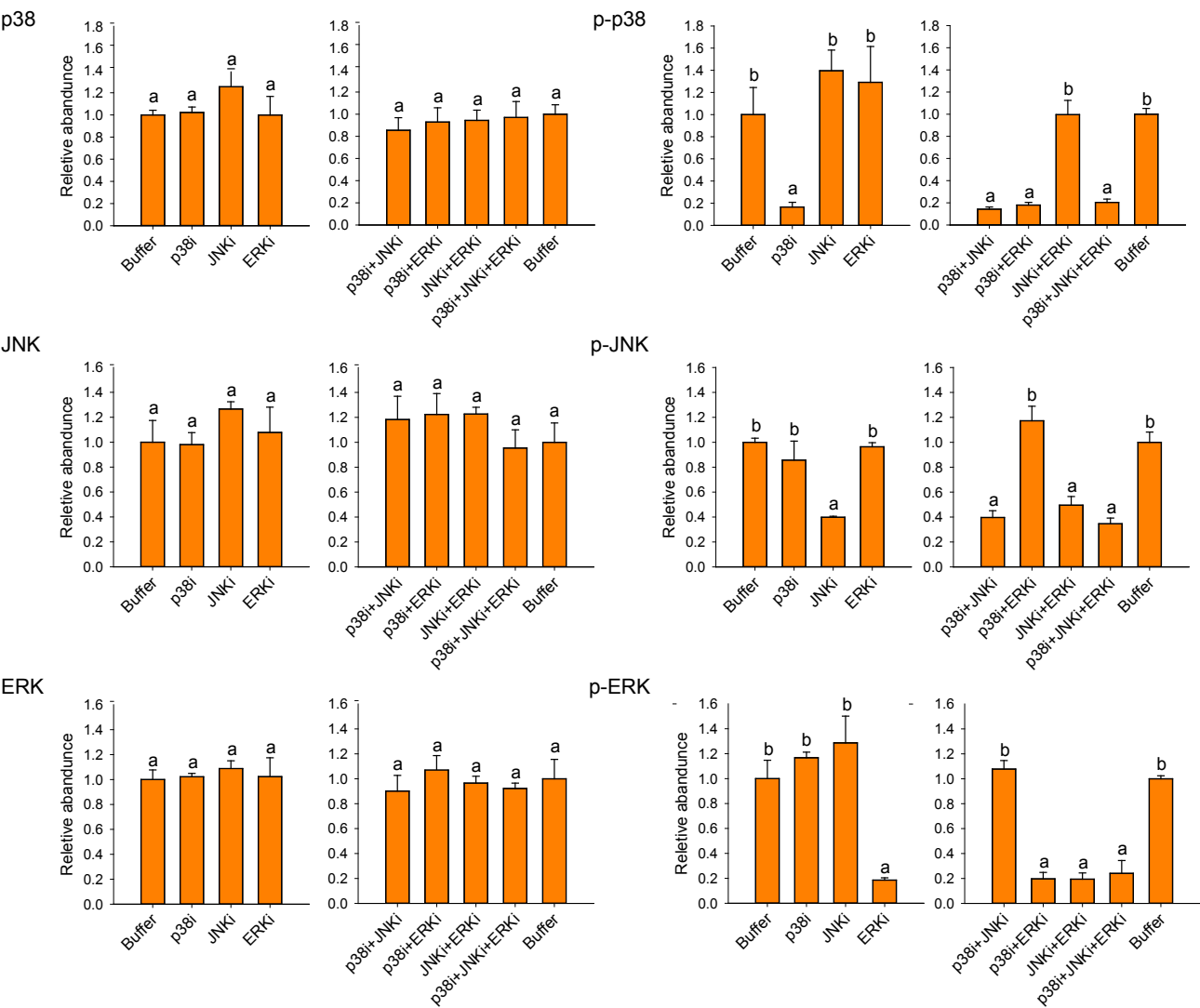

Supplement: S9 Fig — Western blot assays were analyzed with ImageJ 1.51. Data in figures show means and standard errors from three biological replicates. Different letters indicate significant differences between different treatments (P < 0.05; Duncan’s test; n = 3). (PDF) [file ppat.1009917.s009.pdf]
