# Supplementary material for: The regulation landscape of MAPK signaling cascade for thwarting Bacillus thuringiensis infection in an insect host
Source: PLoS Pathog. 2021 Sep 8;17(9):e1009917. doi: 10.1371/journal.ppat.1009917 (PMC8452011; doi:10.1371/journal.ppat.1009917)
Supplement: S3 Fig — Values in each rectangle represent the percent identity of pairs of MAPK cascade kinases. Gene names are at the left and across the top. Percentage identity for each comparison is color-coded according to the gradient value at the bottom. (PDF) [file ppat.1009917.s003.pdf]

S3 Fig

Proteins	MAP4K3	MAP4K4
MAP4K3	100.0%	
MAP4K4	7.9%	100.0%

Proteins	MAP3K4	MAP3K7	MAP3K10	MAP3K12	MAP3K15	Raf	TAO
MAP3K4	100.0%						
MAP3K7	5.0%	100.0%					
MAP3K10	7.1%	13.0%	100.0%				
MAP3K12	4.2%	16.1%	13.2%	100.0%			
MAP3K15	9.2%	8.7%	8.0%	6.6%	100.0%		
Raf	4.6%	9.7%	7.6%	8.0%	6.9%	100.0%	
TAO	6.9%	11.1%	8.2%	7.5%	7.2%	8.1%	100.0%

Proteins	MAP2K1	MAP2K4	MAP2K6	MAP2K7
MAP2K1	100.0%			
MAP2K4	26.6%	100.0%		
MAP2K6	26.8%	38.3%	100.0%	
MAP2K7	14.8%	22.8%	21.5%	100.0%

Proteins	p38	JNK	ERK	MAPK15
p38	100.0%			
JNK	43.8%	100.0%		
ERK	43.9%	33.2%	100.0%	
MAPK15	21.1%	20.3%	24.2%	100.0%

Sequence similarity

<10%	>10%	>20%	>30%	>40%	>50%	>80%	100%
