# Supplementary material for: The regulation landscape of MAPK signaling cascade for thwarting Bacillus thuringiensis infection in an insect host
Source: PLoS Pathog. 2021 Sep 8;17(9):e1009917. doi: 10.1371/journal.ppat.1009917 (PMC8452011; doi:10.1371/journal.ppat.1009917)
Supplement: S1 Table — (DOCX) [file ppat.1009917.s010.docx]

**S1 Table. Genome-wide characterization of the MAPK cascade genes in *P. xylostella*.**

| Genes* | GenBank no. | DBM-DB ID | Scaffold locus (Strand) | Nucleotide features | | Protein features | | |
| --- | --- | --- | --- | --- | --- | --- | --- | --- |
|  |  |  |  | CDS (bp) | Exons^¶^ | Size (aa) | pI/Mw (kDa) | Kinase domain |
| PxMAP4K3 | MN211342 | Px016883^§^ | **85:**42226-74761 (-) | 3249 | 21 | 1142 | 7.96/127.94 | cd06613 |
|  |  | Px014692^§^ | **650:**82946-83981 (-) |  |  |  |  |  |
|  |  | Px013811 | **589:**86786-91735 (+) |  |  |  |  |  |
| PxMAP4K4^†^ | KM507871 | Px002422 | **137:**618079-633773 (-) | 3882 | 20 | 1293 | 9.22/143.17 | cd06608 |
| PxMAP3K4 | MN211343 | Px001582^§^ | **122:**6147-30441 (-) | 4290 | 34 | 1429 | 6.58/160.28 | cd06626 |
|  |  | Px001581^§^ | **122:**983-5697 (-) |  |  |  |  |  |
|  |  | Px004037 | **164:**309685-340060 (-) |  |  |  |  |  |
| PxMAP3K7 | MN211344 | Px002003^§^ | **13:**826348-853524 (+) | 2151 | 10 | 716 | 5.32/81.39 | cd14058 |
|  |  | Px002004^§^ | **13:**865417-867934 (+) |  |  |  |  |  |
| PxMAP3K10 | MN211345 | Px016779^§^ | **83:**856444-891751 (+) | 2967 | 11 | 992 | 6.70/110.40 | cd14061 |
|  |  | Px016780^§^ | **83:**894879-896568 (+) |  |  |  |  |  |
|  |  | Px015735^§^ | **735:**34806-46315 (-) |  |  |  |  |  |
|  |  | Px015734^§^ | **735:**28795-31393 (-) |  |  |  |  |  |
| PxMAP3K12 | MN211346 | Px016598^§^ | **81:**456143-459303 (+) | 1722 | 7 | 573 | 5.38/64.89 | cd14059 |
|  |  | Px016599^§^ | **81:**460347-461391 (+) |  |  |  |  |  |
|  |  | Px016600^§^ | **81:**465952-473774 (+) |  |  |  |  |  |
| PxMAP3K15 | MN211347 | Px001828 | **127:**133382-157674 (+) | 4155 | 25 | 1384 | 5.70/156.48 | cd06624 |
| PxRaf | MN211348 | Px013098 | **527:**89918-117209 (+) | 2208 | 12 | 735 | 8.21/83.56 | cd14062 |
|  |  | Px015389 | **709:**19746-25667 (-) |  |  |  |  |  |
|  |  | Px014045 | **60:**660009-670298 (-) |  |  |  |  |  |
| PxTAO | MN211349 | Px014790 | **66:**376597-397694 (-) | 2979 | 19 | 992 | 8.43/114.81 | cd06607 |
| PxMAP2K1^‡^ | MN211350 | N/A | N/A | 1212 | 8 | 403 | 6.57/44.67 | cd06615 |
| PxMAP2K4 | MN211351 | Px008253 | **295:**195515-201535 (-) | 1239 | 8 | 412 | 8.90/46.30 | cd06616 |
| PxMAP2K6 | MN211352 | Px009355 | **33:**1127090-1128348 (-) | 1011 | 3 | 336 | 6.46/38.13 | cd06617 |
| PxMAP2K7 | MN211353 | Px011704 | **449:**62294-69832 (+) | 2025 | 15 | 674 | 9.67/74.32 | cd06618 |
| Pxp38 | MN211354 | Px015291 | **7:**2252706-2257495 (+) | 1080 | 9 | 359 | 5.93/41.47 | cd07851 |
| PxJNK | MN211355 | Px016832 | **84:**747732-762514 (+) | 1179 | 8 | 392 | 6.31/44.53 | cd07850 |
|  |  | Px016566 | **802:**20525-30478 (-) |  |  |  |  |  |
| PxERK | MN211356 | Px000138 | **10:**914634-949885 (-) | 1092 | 8 | 363 | 5.86/41.72 | cd07849 |
|  |  | Px014553 | **64:**460853-481146 (+) |  |  |  |  |  |
| PxMAPK15 | MN211357 | Px013714 | **58:**72871-97262 (-) | 1656 | 7 | 551 | 9.71/61.91 | cd07852 |
|  |  | Px012756 | **507:**362897-374955 (+) |  |  |  |  |  |

*All the identified *P. xylostella* MAPK cascade genes in this study are named based on genomic annotation and kinase domain.

^†^*PxMAP4K4* was cloned previously (30).

^‡^*PxMAP2K1* has not been found in the *P. xylostella* current genome database.

^§^These sequences in DBM-DB need be assembled as corresponding gene. For *PxMAP3K10*, Px016779 and Px016780 can be assembled as one sequence, while Px015735 and Px015734 can be assembled as another.

^¶^For *PxMAP4K3* and *PxMAP2K1*, the number of exons were validated by DBM fosmid contigs.
