# Supplementary material for: The regulation landscape of MAPK signaling cascade for thwarting Bacillus thuringiensis infection in an insect host
Source: PLoS Pathog. 2021 Sep 8;17(9):e1009917. doi: 10.1371/journal.ppat.1009917 (PMC8452011; doi:10.1371/journal.ppat.1009917)
Supplement: S2 Table — (DOCX) [file ppat.1009917.s011.docx]

**S2 Table. List of the current available MAPK cascade genes in different species.**

| Gene names | Species | Source* | Gene ID^†^ | Size (aa) | Sequence quality | Kinase domain | | Used in this study^‡^ |
| --- | --- | --- | --- | --- | --- | --- | --- | --- |
|  |  |  |  |  |  | ID | Location (aa) |  |
| HsMAP4K1 | *Homo sapiens* | GenBank | NP_009112 | 833 | Complete | cd06613 | 16–274 | Yes |
| HsMAP4K2 | *Homo sapiens* | GenBank | NP_004570 | 820 | Complete | cd06613 | 16–272 | Yes |
| HsMAP4K3 | *Homo sapiens* | GenBank | NP_003609 | 894 | Complete | cd06613 | 15–273 | Yes |
| HsMAP4K4 | *Homo sapiens* | GenBank | NP_001229488 | 1239 | Complete | cl21453 | 8–289 | Yes |
| HsMAP4K5 | *Homo sapiens* | GenBank | NP_006566 | 846 | Complete | cd06646 | 10–277 | Yes |
| HsMAP4K6 | *Homo sapiens* | GenBank | NP_722549 | 1332 | Complete | cd06636 | 8–289 | Yes |
| HsMAP3K1 | *Homo sapiens* | GenBank | NP_005912 | 1512 | Complete | cd06630 | 1242–1509 | Yes |
| HsMAP3K2 | *Homo sapiens* | GenBank | NP_006600 | 619 | Complete | cd06652 | 353–616 | Yes |
| HsMAP3K3 | *Homo sapiens* | GenBank | NP_976226 | 657 | Complete | cd06625 | 393–652 | Yes |
| HsMAP3K4 | *Homo sapiens* | GenBank | NP_005913 | 1608 | Complete | cd06626 | 1342–1601 | Yes |
| HsMAP3K5 | *Homo sapiens* | GenBank | NP_005914 | 1372 | Complete | cd06624 | 671–938 | Yes |
| HsMAP3K6 | *Homo sapiens* | GenBank | NP_004663 | 1288 | Complete | cd06624 | 639–906 | Yes |
| HsMAP3K7 | *Homo sapiens* | GenBank | NP_663304 | 606 | Complete | cd14058 | 42–292 | Yes |
| HsMAP3K8 | *Homo sapiens* | GenBank | NP_005195 | 467 | Complete | cd13995 | 133–388 | Yes |
| HsMAP3K9 | *Homo sapiens* | GenBank | NP_149132 | 1188 | Complete | cd14151 | 137–406 | Yes |
| HsMAP3K10 | *Homo sapiens* | GenBank | NP_002437 | 954 | Complete | cd14148 | 103–360 | Yes |
| HsMAP3K11 | *Homo sapiens* | GenBank | NP_002410 | 847 | Complete | cd14147 | 113–379 | Yes |
| HsMAP3K12 | *Homo sapiens* | GenBank | NP_006292 | 859 | Complete | cd14059 | 131–367 | Yes |
| HsMAP3K13 | *Homo sapiens* | GenBank | NP_004712 | 966 | Complete | cd14059 | 174–410 | Yes |
| HsMAP3K14 | *Homo sapiens* | GenBank | NP_003945 | 947 | Complete | cd13991 | 393–660 | Yes |
| HsMAP3K15 | *Homo sapiens* | GenBank | NP_001001671 | 1313 | Complete | cd06624 | 643–908 | Yes |
| HsMAP3K16 | *Homo sapiens* | GenBank | NP_065842 | 1001 | Complete | cd06635 | 2–318 | Yes |
| HsMAP3K17 | *Homo sapiens* | GenBank | NP_057235 | 1235 | Complete | cd06634 | 12–319 | Yes |
| HsMAP3K18 | *Homo sapiens* | GenBank | NP_057365 | 898 | Complete | cd06633 | 2–314 | Yes |
| HsMAP3K19 | *Homo sapiens* | GenBank | NP_079328 | 1328 | Complete | cd06631 | 1059–1324 | Yes |
| HsARAF | *Homo sapiens* | GenBank | NP_001645 | 606 | Complete | cd14150 | 309–573 | Yes |
| HsBRAF | *Homo sapiens* | GenBank | NP_004324 | 766 | Complete | cd14062 | 463–715 | Yes |
| HsRAF1 | *Homo sapiens* | GenBank | NP_002871 | 648 | Complete | cd14149 | 336–618 | Yes |
| HsMOS | *Homo sapiens* | GenBank | NP_005363 | 346 | Complete | cd13979 | 56–338 | Yes |
| HsMAP2K1 | *Homo sapiens* | GenBank | NP_002746 | 393 | Complete | cd06650 | 62–380 | Yes |
| HsMAP2K2 | *Homo sapiens* | GenBank | NP_109587 | 400 | Complete | cd06649 | 66–396 | Yes |
| HsMAP2K3 | *Homo sapiens* | GenBank | NP_659731 | 347 | Complete | cd06617 | 62–344 | Yes |
| HsMAP2K4 | *Homo sapiens* | GenBank | NP_003001 | 399 | Complete | cd06616 | 95–385 | Yes |
| HsMAP2K5 | *Homo sapiens* | GenBank | NP_660143 | 448 | Complete | cd06619 | 164–442 | Yes |
| HsMAP2K6 | *Homo sapiens* | GenBank | NP_002749 | 334 | Complete | cd06617 | 51–333 | Yes |
| HsMAP2K7 | *Homo sapiens* | GenBank | NP_660186 | 419 | Complete | cd06618 | 104–400 | Yes |
| HsMAPK1 | *Homo sapiens* | GenBank | NP_620407 | 360 | Complete | cd07849 | 19–353 | Yes |
| HsMAPK3 | *Homo sapiens* | GenBank | NP_002737 | 379 | Complete | cd07849 | 36–370 | Yes |
| HsMAPK4 | *Homo sapiens* | GenBank | NP_002738 | 587 | Complete | cd07854 | 14–351 | Yes |
| HsMAPK6 | *Homo sapiens* | GenBank | NP_002739 | 721 | Complete | cd07854 | 14–355 | Yes |
| HsMAPK7 | *Homo sapiens* | GenBank | NP_002740 | 816 | Complete | cd07855 | 49–384 | Yes |
| HsMAPK8 | *Homo sapiens* | GenBank | NP_002741 | 384 | Complete | cd07850 | 25–360 | Yes |
| HsMAPK9 | *Homo sapiens* | GenBank | NP_620707 | 382 | Complete | cd07850 | 25–360 | Yes |
| HsMAPK10 | *Homo sapiens* | GenBank | NP_620448 | 464 | Complete | cd07850 | 63–398 | Yes |
| HsMAPK11 | *Homo sapiens* | GenBank | NP_002742 | 364 | Complete | cl21453 | 8–348 | Yes |
| HsMAPK12 | *Homo sapiens* | GenBank | NP_002960 | 367 | Complete | cd07880 | 11–353 | Yes |
| HsMAPK13 | *Homo sapiens* | GenBank | NP_002745 | 365 | Complete | cd07879 | 9–351 | Yes |
| HsMAPK14 | *Homo sapiens* | GenBank | NP_620581 | 360 | Complete | cd07877 | 6–350 | Yes |
| HsMAPK15 | *Homo sapiens* | GenBank | NP_620590 | 544 | Complete | cd07852 | 5–343 | Yes |
| CeGCK | *Caenorhabditis elegans* | GenBank | NP_504721 | 829 | Complete | cd06613 | 14–273 | Yes |
| CeMig15 | *Caenorhabditis elegans* | GenBank | NP_001024971 | 1087 | Complete | cd06608 | 14–288 | Yes |
| CeMTK1 | *Caenorhabditis elegans* | GenBank | NP_491683 | 1418 | Complete | cd06626 | 1124–1388 | Yes |
| CeNSY1 | *Caenorhabditis elegans* | GenBank | NP_001293513 | 1498 | Complete | cd06624 | 655–925 | Yes |
| CeMOM4 | *Caenorhabditis elegans* | GenBank | NP_492620 | 536 | Complete | cd13999 | 57–311 | Yes |
| CeMLK1 | *Caenorhabditis elegans* | GenBank | NP_741537 | 1040 | Complete | cd13999 | 175–453 | Yes |
| CeRaf | *Caenorhabditis elegans* | GenBank | NP_741431 | 855 | Complete | cd14062 | 529–786 | Yes |
| CeSULU | *Caenorhabditis elegans* | GenBank | NP_001022767 | 982 | Complete | cd06607 | 28–291 | Yes |
| CeMEK2 | *Caenorhabditis elegans* | GenBank | NP_491087 | 387 | Complete | cd06615 | 71–380 | Yes |
| CeSEK1 | *Caenorhabditis elegans* | GenBank | NP_509322 | 336 | Complete | cd06617 | 48–331 | Yes |
| CeMKK4 | *Caenorhabditis elegans* | GenBank | NP_509682 | 363 | Complete | cd06616 | 59–347 | Yes |
| CeMEK1 | *Caenorhabditis elegans* | GenBank | NP_001024771 | 347 | Complete | cd06618 | 56–341 | Yes |
| CePMK1 | *Caenorhabditis elegans* | GenBank | NP_501365 | 377 | Complete | cd07851 | 19–361 | Yes |
| CeJNK1 | *Caenorhabditis elegans* | GenBank | NP_001021270 | 463 | Complete | cd07850 | 118–452 | Yes |
| CeMPK1 | *Caenorhabditis elegans* | GenBank | NP_001022583 | 376 | Complete | cd07849 | 22–356 | Yes |
| CeMPK2 | *Caenorhabditis elegans* | GenBank | NP_494946 | 605 | Complete | cd07855 | 54–387 | Yes |
| CeMAPK | *Caenorhabditis elegans* | GenBank | NP_872069 | 418 | Complete | cd07834 | 44–355 | Yes |
| AgMEKK4 | *Anopheles gambiae* | GenBank | XP_312585 | 1550 | Complete | cl21453 | 1262–1520 | Yes |
| AgMEKK5/6 | *Anopheles gambiae* | GenBank | XP_311281 | 1481 | Complete | cd06624 | 609–876 | Yes |
| AgTAK1 | *Anopheles gambiae* | GenBank | XP_003436321 | 604 | Complete | cd14058 | 23–272 | Yes |
| AgMLK2/3 | *Anopheles gambiae* | GenBank | XP_312218 | 1023 | Complete | cd14061 | 108–363 | Yes |
| AgM3K12/13 | *Anopheles gambiae* | GenBank | XP_316502 | 1117 | Complete | cl21453 | 157–394 | Yes |
| AgRaf | *Anopheles gambiae* | GenBank | XP_318144 | 667 | Complete | cd14062 | 362–607 | Yes |
| AgTAO | *Anopheles gambiae* | GenBank | XP_001237504 | 983 | Complete | cd06607 | 22–279 | Yes |
| AgMOS | *Anopheles gambiae* | GenBank | XP_308274 | 428 | Complete | cl21453 | 36–285 | Yes |
| AgMEK1 | *Anopheles gambiae* | GenBank | XP_322064 | 406 | Complete | cd06615 | 91–390 | Yes |
| AgMEK3/6 | *Anopheles gambiae* | GenBank | XP_310813 | 393 | Complete | cl21453 | 57–340 | Yes |
| AgMKK4 | *Anopheles gambiae* | GenBank | XP_314266 | 371 | Complete | cd06616 | 56–346 | Yes |
| AgMAP2K7 | *Anopheles gambiae* | GenBank | XP_321199 | 1165 | Complete | cd06618 | 111–402 | Yes |
| AgERK | *Anopheles gambiae* | GenBank | XP_319983 | 331 | Partial | cd07849 | 11–323 | No |
| AgJNKb | *Anopheles gambiae* | GenBank | XP_310236 | 375 | Complete | cd07850 | 22–358 | Yes |
| AgJNKa | *Anopheles gambiae* | GenBank | XP_307879 | 361 | Partial | cl21453 | 13–348 | Yes |
| Agp38 | *Anopheles gambiae* | GenBank | XP_320380 | 184 | Partial | N/A | N/A | No |
| DmHappyhour | *Drosophila melanogaster* | GenBank | NP_725863 | 1218 | Complete | cd06613 | 25–283 | Yes |
| DmMisshapen | *Drosophila melanogaster* | GenBank | NP_524679 | 1504 | Complete | cd06608 | 25–296 | Yes |
| DmMEKK1 | *Drosophila melanogaster* | GenBank | NP_732373 | 1571 | Complete | cd06626 | 1281–1542 | Yes |
| DmPK92B | *Drosophila melanogaster* | GenBank | NP_477089 | 1363 | Complete | cd06624 | 576–843 | Yes |
| DmTAK1 | *Drosophila melanogaster* | GenBank | NP_524080 | 678 | Complete | cd14058 | 25–275 | Yes |
| DmSlipper | *Drosophila melanogaster* | GenBank | NP_001188558 | 1155 | Complete | cd14061 | 134–389 | Yes |
| DmWallenda | *Drosophila melanogaster* | GenBank | NP_649137 | 977 | Complete | cd14059 | 167–403 | Yes |
| DmPolehole | *Drosophila melanogaster* | GenBank | NP_525047 | 739 | Complete | cd14062 | 435–687 | Yes |
| DmTAO | *Drosophila melanogaster* | GenBank | NP_728267 | 1039 | Complete | cd06607 | 25–282 | Yes |
| Dmmos | *Drosophila melanogaster* | GenBank | NP_610817 | 364 | Complete | cd13979 | 19–298 | Yes |
| DmDSOR | *Drosophila melanogaster* | GenBank | NP_511098 | 396 | Complete | cd06615 | 85–382 | Yes |
| DmLicorne | *Drosophila melanogaster* | GenBank | NP_477162 | 334 | Complete | cd06617 | 44–326 | Yes |
| DmMKK4 | *Drosophila melanogaster* | GenBank | NP_477353 | 424 | Complete | cd06616 | 115–405 | Yes |
| DmHemipterous | *Drosophila melanogaster* | GenBank | NP_727661 | 1178 | Complete | cd06618 | 181–476 | Yes |
| DmRolled | *Drosophila melanogaster* | GenBank | NP_001015122 | 376 | Complete | cd07849 | 32–366 | Yes |
| Dmp38a | *Drosophila melanogaster* | GenBank | NP_477163 | 366 | Complete | cd07851 | 9–354 | Yes |
| Dmp38b | *Drosophila melanogaster* | GenBank | NP_477361 | 365 | Complete | cd07851 | 8–353 | Yes |
| Dmp38c | *Drosophila melanogaster* | GenBank | NP_996277 | 356 | Complete | cd07851 | 4–349 | No |
| DmBasket | *Drosophila melanogaster* | GenBank | NP_723541 | 372 | Complete | cd07850 | 23–359 | Yes |
| DmERK7 | *Drosophila melanogaster* | GenBank | NP_727335 | 916 | Complete | cd07852 | 17–358 | Yes |
| BmMAP4K3 | *Bombyx mori* | GenBank | XP_012552948 | 1110 | Complete | cd06613 | 20–279 | Yes |
| BmMAP4K4 | *Bombyx mori* | GenBank | XP_004928503 | 1380 | Complete | cd06608 | 21–292 | Yes |
| BmMAP3K4 | *Bombyx mori* | GenBank | XP_012546186 | 1470 | Complete | cd06626 | 1193–1450 | No |
| BmMAP3K7 | *Bombyx mori* | GenBank | XP_004931740 | 710 | Complete | cd14058 | 33–283 | Yes |
| BmMAP3K10 | *Bombyx mori* | GenBank | XP_004932970+XP_012545643 | 948 | Partial | cd14061 | 98–356 | Yes |
| BmMAP3K12 | *Bombyx mori* | GenBank | XP_012551431 | 470 | Complete | cl21453 | 22–258 | Yes |
| BmMAP3K15 | *Bombyx mori* | GenBank | NP_001188511 | 1346 | Complete | cd06624 | 614–881 | Yes |
| BmRaf | *Bombyx mori* | GenBank | NP_001189459 | 700 | Complete | cd14062 | 383–635 | Yes |
| BmTAO | *Bombyx mori* | GenBank | XP_012548250 | 1005 | Complete | cd06607 | 23–280 | Yes |
| BmMAP2K1 | *Bombyx mori* | GenBank | NP_001036922 | 404 | Complete | cd06615 | 89–388 | Yes |
| BmMAP2K4 | *Bombyx mori* | GenBank | XP_004927053 | 353 | Complete | cd06616 | 43–331 | Yes |
| BmMAP2K6 | *Bombyx mori* | GenBank | XP_004921977 | 334 | Complete | cd06617 | 45–329 | Yes |
| BmMAP2K7 | *Bombyx mori* | GenBank | NP_001243912 | 409 | Complete | cd06618 | 29–325 | No |
| Bmp38 | *Bombyx mori* | GenBank | NP_001036996 | 360 | Complete | cd07851 | 4–346 | Yes |
| BmJNK | *Bombyx mori* | GenBank | NP_001103396 | 396 | Complete | cd07850 | 34–370 | Yes |
| BmERK | *Bombyx mori* | GenBank | NP_001036921 | 364 | Complete | cd07849 | 22–356 | Yes |
| BmMAPK15 | *Bombyx mori* | GenBank | XP_004927281 | 531 | Complete | cd07852 | 30–372 | Yes |
| MsMAP4K3 | *Manduca sexta* | Manduca Base | Msex2.12722+Msex2.14253 | 142+129 | Partial | N/A | N/A | No |
| MsMAP4K4 | *Manduca sexta* | Manduca Base | Msex2.06876 | 1087 | Complete | cd06608 | 22–293 | Yes |
| MsMAP3K4 | *Manduca sexta* | Manduca Base | Msex2.04243 | 1457 | Complete | cl21453 | 1183–1440 | Yes |
| MsMAP3K7 | *Manduca sexta* | Manduca Base | Msex2.02602 | 722 | Complete | cd14058 | 29–279 | Yes |
| MsMAP3K10 | *Manduca sexta* | Manduca Base | Msex2.07169 | 241 | Partial | N/A | N/A | No |
| MsMAP3K12 | *Manduca sexta* | Manduca Base | Msex2.02840 | 531 | Complete | cl21453 | 78–314 | Yes |
| MsMAP3K15 | *Manduca sexta* | Manduca Base | Msex2.05568 | 1310 | Complete | cd06624 | 614–881 | Yes |
| MsRaf | *Manduca sexta* | Manduca Base | Msex2.08712 | 740 | Complete | cd14062 | 419–676 | Yes |
| MsTAO | *Manduca sexta* | Manduca Base | Msex2.03616 | 992 | Complete | cd06607 | 23–280 | Yes |
| MsMAP2K1 | *Manduca sexta* | Manduca Base | Msex2.00725 | 403 | Complete | cd06615 | 88–387 | Yes |
| MsMAP2K4 | *Manduca sexta* | Manduca Base | Msex2.02705 | 409 | Complete | cd06616 | 99–387 | Yes |
| MsMAP2K6 | *Manduca sexta* | Manduca Base | Msex2.07813 | 241 | Partial | N/A | N/A | No |
| Msp38 | *Manduca sexta* | Manduca Base | Msex2.09629 | 360 | Complete | cd07851 | 4–346 | Yes |
| MsJNK | *Manduca sexta* | Manduca Base | Msex2.05111 | 368 | Partial | cd07850 | 74–356 | No |
| MsERK | *Manduca sexta* | Manduca Base | Msex2.04292 | 364 | Complete | cd07849 | 22–355 | Yes |
| MsMAPK15 | *Manduca sexta* | Manduca Base | Msex2.11041 | 537 | Complete | cd07852 | 28–370 | Yes |
| DpMAP4K3 | *Danaus plexippus* | GenBank | OWR48434 | 721 | Partial | N/A | N/A | No |
| DpMAP4K4 | *Danaus plexippus* | GenBank | OWR46460 | 1359 | Complete | cd06636 | 12–292 | Yes |
| DpMAP3K4 | *Danaus plexippus* | GenBank | OWR52915 | 1406 | Complete | cd06626 | 1130–1389 | Yes |
| DpMAP3K7 | *Danaus plexippus* | GenBank | OWR51860 | 609 | Complete | cd14058 | 30–280 | Yes |
| DpMAP3K10 | *Danaus plexippus* | GenBank | OWR52681+OWR52680 | 288+441 | Partial | N/A | N/A | No |
| DpMAP3K12 | *Danaus plexippus* | GenBank | OWR50434 | 529 | Complete | cl21453 | 4–240 | Yes |
| DpASK | *Danaus plexippus* | GenBank | OWR42072 | 1358 | Complete | cd06624 | 615–879 | Yes |
| DpRaf | *Danaus plexippus* | GenBank | OWR49271 | 849 | Complete | cd14062 | 532–785 | Yes |
| DpTAO | *Danaus plexippus* | GenBank | OWR54956 | 983 | Complete | cd06607 | 56–313 | Yes |
| DpMAP2K1 | *Danaus plexippus* | GenBank | OWR53027 | 403 | Complete | cd06615 | 85–384 | Yes |
| DpMAP2K4 | *Danaus plexippus* | GenBank | OWR47007 | 409 | Complete | cd06616 | 99–387 | Yes |
| DpMAP2K6 | *Danaus plexippus* | GenBank | OWR43290 | 338 | Partial | N/A | N/A | No |
| DpMAP2K7 | *Danaus plexippus* | GenBank | OWR50126 | 682 | Complete | cd06618 | 80–373 | Yes |
| Dpp38 | *Danaus plexippus* | GenBank | OWR52323 | 360 | Complete | cd07851 | 4–346 | Yes |
| DpJNK | *Danaus plexippus* | GenBank | OWR45290 | 449 | Complete | cd07850 | 32–368 | Yes |
| DpERK | *Danaus plexippus* | GenBank | OWR45948 | 166 | Partial | N/A | N/A | No |
| DpMAPK15 | *Danaus plexippus* | GenBank | OWR49765 | 490 | Complete | cd07852 | 4–346 | Yes |
| ApMAP4K3 | *Acyrthosiphon pisum* | GenBank | XP_001942639 | 881 | Complete | cd06613 | 13–271 | Yes |
| ApMAP4K4 | *Acyrthosiphon pisum* | GenBank | XP_016662496 | 1199 | Complete | cd06618 | 24–295 | Yes |
| ApMAP3K4 | *Acyrthosiphon pisum* | GenBank | XP_008180859 | 1195 | Complete | cd06626 | 905–1167 | Yes |
| ApMAP3K7 | *Acyrthosiphon pisum* | GenBank | XP_001944457 | 424 | Complete | cd14058 | 28–276 | Yes |
| ApMAP3K10 | *Acyrthosiphon pisum* | GenBank | XP_008190024 | 1110 | Complete | cd14061 | 152–409 | Yes |
| ApMAP3K13 | *Acyrthosiphon pisum* | GenBank | XP_016658937 | 729 | Complete | cd14059 | 125–361 | Yes |
| ApMAP3K15 | *Acyrthosiphon pisum* | GenBank | XP_016662593 | 1376 | Complete | cd06624 | 615–882 | Yes |
| ApRaf | *Acyrthosiphon pisum* | GenBank | XP_001952293 | 654 | Complete | cd14062 | 351–602 | Yes |
| ApTAO | *Acyrthosiphon pisum* | GenBank | XP_001950417 | 902 | Complete | cd06607 | 26–283 | Yes |
| ApMAP2K1 | *Acyrthosiphon pisum* | GenBank | XP_001948295 | 401 | Complete | cd06615 | 78–383 | Yes |
| ApMAP2K4 | *Acyrthosiphon pisum* | GenBank | XP_016658524 | 442 | Complete | cd06616 | 138–426 | Yes |
| ApMAP2K6 | *Acyrthosiphon pisum* | GenBank | XP_001952170 | 338 | Complete | cd06617 | 48–330 | Yes |
| ApMAP2K7 | *Acyrthosiphon pisum* | GenBank | XP_008180171 | 630 | Complete | cd06618 | 89–378 | Yes |
| App38 | *Acyrthosiphon pisum* | GenBank | XP_008186810 | 352 | Complete | cd07851 | 3–345 | Yes |
| ApJNK | *Acyrthosiphon pisum* | GenBank | XP_016657935 | 397 | Complete | cd07850 | 41–377 | Yes |
| ApERK | *Acyrthosiphon pisum* | GenBank | XP_001952106 | 361 | Complete | cd07849 | 22–354 | Yes |
| TcMAP4K3 | *Tribolium castaneum* | GenBank | XP_015833881 | 924 | Complete | cd06613 | 18–276 | Yes |
| TcMAP4K4 | *Tribolium castaneum* | GenBank | XP_015835920 | 1251 | Complete | cd06608 | 21–292 | Yes |
| TcMAP3K4 | *Tribolium castaneum* | GenBank | XP_008201376 | 1250 | Complete | cd06626 | 981–1243 | Yes |
| TcMAP3K7 | *Tribolium castaneum* | GenBank | XP_968547 | 511 | Complete | cd14058 | 29–277 | Yes |
| TcMAP3K11 | *Tribolium castaneum* | GenBank | XP_015834889 | 1011 | Complete | cd14061 | 129–386 | Yes |
| TcMAP3K13 | *Tribolium castaneum* | GenBank | XP_015833520 | 843 | Complete | cl21453 | 139–376 | Yes |
| TcMAP3K15 | *Tribolium castaneum* | GenBank | XP_008201566 | 1276 | Complete | cd06624 | 603–870 | Yes |
| TcRaf | *Tribolium castaneum* | GenBank | XP_008198803 | 704 | Complete | cd14062 | 397–649 | Yes |
| Tcmos | *Tribolium castaneum* | GenBank | XP_008201578 | 341 | Complete | cl21453 | 93–326 | Yes |
| TcTAO | *Tribolium castaneum* | GenBank | XP_015836277 | 790 | Complete | cl21453 | 6–316 | Yes |
| TcMAP2K1 | *Tribolium castaneum* | GenBank | XP_973216 | 386 | Complete | cd06615 | 80–377 | Yes |
| TcMAP2K4 | *Tribolium castaneum* | GenBank | XP_015838337 | 412 | Complete | cd06616 | 104–396 | Yes |
| TcMAP2K6 | *Tribolium castaneum* | GenBank | XP_008197122 | 334 | Complete | cd06617 | 47–329 | Yes |
| TcMAP2K7 | *Tribolium castaneum* | GenBank | XP_008201625 | 662 | Complete | cd06618 | 56–349 | Yes |
| Tcp38 | *Tribolium castaneum* | GenBank | XP_008193438 | 354 | Complete | cd07851 | 6–344 | Yes |
| TcJNK | *Tribolium castaneum* | GenBank | XP_008197492 | 412 | Complete | cd07850 | 44–380 | Yes |
| TcERK | *Tribolium castaneum* | GenBank | XP_966833 | 372 | Complete | cd07849 | 22–355 | Yes |
| TcMAPK15 | *Tribolium castaneum* | GenBank | XP_973435 | 488 | Complete | cd07852 | 11–351 | Yes |
| AmMAP4K3 | *Apis mellifera* | GenBank | XP_006558871 | 941 | Complete | cd06613 | 20–278 | Yes |
| AmMAP4K4 | *Apis mellifera* | GenBank | XP_006566210 | 1345 | Complete | cd06608 | 21–292 | Yes |
| AmMAP3K4 | *Apis mellifera* | GenBank | XP_392650 | 1323 | Complete | cl21453 | 1044–1306 | Yes |
| AmMAP3K7 | *Apis mellifera* | GenBank | XP_397248 | 548 | Complete | cd14058 | 28–277 | Yes |
| AmMAP3K11 | *Apis mellifera* | GenBank | XP_016770532 | 1194 | Complete | cd14061 | 204–461 | Yes |
| AmMAP3K15 | *Apis mellifera* | GenBank | XP_006563423 | 1359 | Complete | cd06624 | 618–885 | Yes |
| AmRaf | *Apis mellifera* | GenBank | XP_396892 | 715 | Complete | cd14062 | 408–660 | Yes |
| AmTAO | *Apis mellifera* | GenBank | XP_006572321 | 897 | Complete | cd06607 | 26–283 | Yes |
| Ammos | *Apis mellifera* | GenBank | NP_001011644 | 316 | Complete | cd13979 | 63–312 | Yes |
| AmMAP2K1 | *Apis mellifera* | GenBank | XP_393416 | 414 | Complete | cd06615 | 85–399 | Yes |
| AmMAP2K3 | *Apis mellifera* | GenBank | XP_006562044 | 331 | Complete | cl21453 | 46–326 | Yes |
| AmMAP2K4 | *Apis mellifera* | GenBank | XP_006563748 | 436 | Complete | cd06616 | 130–420 | Yes |
| AmMAP2K7 | *Apis mellifera* | GenBank | XP_006558043 | 764 | Complete | cd06618 | 84-388 | Yes |
| Amp38 | *Apis mellifera* | GenBank | XP_003250351 | 360 | Complete | cd07851 | 4–346 | Yes |
| AmERK | *Apis mellifera* | GenBank | XP_393029 | 365 | Complete | cd07849 | 21–354 | Yes |
| AmJNK | *Apis mellifera* | GenBank | XP_006557300 | 527 | Complete | cd07850 | 169–505 | Yes |
| AmMAPK15 | *Apis mellifera* | GenBank | XP_006567336 | 526 | Complete | cd07852 | 13–354 | Yes |
| TuMAP4K3 | *Tetranychus urticae* | GenBank | XP_015792503 | 1003 | Complete | cd06613 | 10–268 | Yes |
| TuMAP4K4 | *Tetranychus urticae* | GenBank | XP_015782680 | 1318 | Complete | cd06608 | 22–293 | Yes |
| TuMAP3K7 | *Tetranychus urticae* | GenBank | XP_015784617 | 518 | Complete | cl21453 | 45–288 | Yes |
| TuMAP3K9 | *Tetranychus urticae* | GenBank | XP_015794661 | 909 | Complete | cl21453 | 132–379 | Yes |
| TuMAP3K13 | *Tetranychus urticae* | GenBank | XP_015794469 | 557 | Complete | cl21453 | 148–383 | Yes |
| TuMAP3K15 | *Tetranychus urticae* | GenBank | XP_015795867 | 1316 | Complete | cl21453 | 600–867 | Yes |
| TuRaf | *Tetranychus urticae* | GenBank | XP_015788884 | 665 | Complete | cd14062 | 360–612 | Yes |
| Tumos | *Tetranychus urticae* | GenBank | XP_015789296 | 308 | Complete | cd13979 | 18–289 | Yes |
| TuTAO | *Tetranychus urticae* | GenBank | XP_015789434 | 893 | Complete | cd06607 | 23–281 | Yes |
| TuMAP2K1 | *Tetranychus urticae* | GenBank | XP_015789239 | 431 | Complete | cd06615 | 73–400 | Yes |
| TuMAP2K6 | *Tetranychus urticae* | GenBank | XP_015793867 | 379 | Complete | cl21453 | 69–376 | Yes |
| TuMAP2K7 | *Tetranychus urticae* | GenBank | XP_015794871 | 419 | Complete | cl21453 | 111–415 | Yes |
| Tup38 | *Tetranychus urticae* | GenBank | XP_015794838 | 351 | Complete | cd07851 | 7–349 | Yes |
| TuERK | *Tetranychus urticae* | GenBank | XP_015782920 | 358 | Complete | cd07849 | 10–344 | Yes |
| TuJNK | *Tetranychus urticae* | GenBank | XP_015784065 | 408 | Complete | cd07850 | 39–375 | Yes |
| PhMAP4K3 | *Pediculus humanus corporis* | GenBank | XP_002424548 | 928 | Complete | cd06613 | 20–278 | Yes |
| PhMAP4K4 | *Pediculus humanus corporis* | GenBank | XP_002423648 | 1210 | Complete | cl21453 | 12–248 | Yes |
| PhMAP3K7 | *Pediculus humanus corporis* | GenBank | XP_002424288 | 349 | Partial | cd14058 | 28–276 | Yes |
| PhMAP3K10 | *Pediculus humanus corporis* | GenBank | XP_002425995 | 758 | Complete | cd14061 | 205–462 | Yes |
| PhMAP3K12 | *Pediculus humanus corporis* | GenBank | XP_002432404 | 857 | Complete | cl21453 | 94–330 | Yes |
| PhMAP3K15 | *Pediculus humanus corporis* | GenBank | XP_002432644 | 1382 | Complete | cd06624 | 629–896 | Yes |
| PhRaf | *Pediculus humanus corporis* | GenBank | XP_002430295 | 689 | Complete | cd14062 | 374–626 | Yes |
| Phmos | *Pediculus humanus corporis* | GenBank | XP_002432409 | 627 | Complete | cd13979 | 115–360  394–625 | Yes |
| PhTAO | *Pediculus humanus corporis* | GenBank | XP_002426013 | 903 | Complete | cd06607 | 26–283 | Yes |
| PhMAP2K1 | *Pediculus humanus corporis* | GenBank | XP_002428982 | 400 | Complete | cd06615 | 87–387 | Yes |
| PhMAP2K4 | *Pediculus humanus corporis* | GenBank | XP_002425403 | 372 | Complete | cd06616 | 66–356 | Yes |
| PhMAP2K6 | *Pediculus humanus corporis* | GenBank | XP_002428268 | 337 | Complete | cd06617 | 49–330 | Yes |
| PhMAP2K7 | *Pediculus humanus corporis* | GenBank | XP_002430642 | 772 | Complete | cd06618 | 100–394 | Yes |
| Php38 | *Pediculus humanus corporis* | GenBank | XP_002428549 | 357 | Complete | cd07851 | 4–346 | Yes |
| PhJNK | *Pediculus humanus corporis* | GenBank | XP_002424379 | 436 | Complete | cd07850 | 64–400 | Yes |
| PhERK | *Pediculus humanus corporis* | GenBank | XP_002424626 | 117 | Partial | N/A | N/A | No |
| PhMAPK15 | *Pediculus humanus* | GenBank | XP_002425485 | 562 | Complete | cd07852 | 17–355 | Yes |
| NvMAP4K3 | *Nasonia vitripennis* | GenBank | XP_008209700 | 951 | Complete | cd06613 | 21–279 | Yes |
| NvMAP4K4 | *Nasonia vitripennis* | GenBank | XP_001599608 | 978 | Complete | cd06608 | 21–292 | Yes |
| NvMAP3K4 | *Nasonia vitripennis* | GenBank | XP_001602559 | 1325 | Complete | cd06626 | 1049–1310 | Yes |
| NvMAP3K7 | *Nasonia vitripennis* | GenBank | XP_001604249 | 533 | Complete | cd14058 | 25–274 | Yes |
| NvMAP3K13 | *Nasonia vitripennis* | GenBank | XP_008212317 | 900 | Complete | cl21453 | 119–355 | Yes |
| NvMAP3K15 | *Nasonia vitripennis* | GenBank | XP_016842430 | 1360 | Complete | cd06624 | 625–892 | Yes |
| NvRaf | *Nasonia vitripennis* | GenBank | XP_001605107 | 789 | Complete | cd14062 | 482–734 | Yes |
| Nvmos | *Nasonia vitripennis* | GenBank | XP_016844316 | 333 | Complete | cl21453 | 81–320 | Yes |
| NvTAO | *Nasonia vitripennis* | GenBank | XP_003427006 | 973 | Complete | cd06607 | 104–361 | Yes |
| NvMAP2K1 | *Nasonia vitripennis* | GenBank | XP_008217848 | 427 | Complete | cd06615 | 85–412 | Yes |
| NvMAP2K4 | *Nasonia vitripennis* | GenBank | XP_001603456 | 441 | Complete | cd06616 | 135–425 | Yes |
| NvMAP2K6 | *Nasonia vitripennis* | GenBank | XP_008206089 | 338 | Complete | cd06617 | 48–330 | Yes |
| NvMAP2K7 | *Nasonia vitripennis* | GenBank | XP_001604642 | 776 | Complete | cd06618 | 101–403 | Yes |
| Nvp38 | *Nasonia vitripennis* | GenBank | NP_001136337 | 356 | Complete | cd07851 | 4–346 | Yes |
| NvJNK | *Nasonia vitripennis* | GenBank | XP_016837523 | 397 | Complete | cd07850 | 32–375 | Yes |
| NvERK | *Nasonia vitripennis* | GenBank | XP_016837193 | 366 | Complete | cd07849 | 22–355 | Yes |
| NvMAPK15 | *Nasonia vitripennis* | GenBank | XP_008204877 | 705 | Complete | cd07852 | 14–355 | Yes |
| RpMAP4K3 | *Rhodnius prolixus* | VectorBase | RPRC004886-PA | 1208 | Complete | cd06613 | 21–278 | Yes |
| RpMAP4K4 | *Rhodnius prolixus* | VectorBase | RPRC006941-PA | 1280 | Partial | N/A | N/A | No |
| RpMAP3K4 | *Rhodnius prolixus* | VectorBase | RPRC004150-PA | 1038 | Complete | cl21453 | 750–1011 | Yes |
| RpMAP3K7 | *Rhodnius prolixus* | VectorBase | RPRC014191-PA+RPRC014214-PA | 432 | Partial | cd14058 | 28–279 | Yes |
| RpMAP3K10 | *Rhodnius prolixus* | VectorBase | RPRC008784-PA | 538 | Partial | cd14061 | 84–341 | Yes |
| RpMAP3K15 | *Rhodnius prolixus* | VectorBase | RPRC001119-PA | 796 | Partial | N/A | N/A | No |
| RpRaf | *Rhodnius prolixus* | VectorBase | RPRC004230-PA | 682 | Complete | cd14062 | 377–628 | Yes |
| Rpmos | *Rhodnius prolixus* | VectorBase | RPRC013487-PA | 401 | Complete | cd13979 | 88–316 | Yes |
| RpMAP2K1 | *Rhodnius prolixus* | VectorBase | RPRC002914-PA | 373 | Partial | cd06615 | 44–360 | Yes |
| RpMAP2K4 | *Rhodnius prolixus* | VectorBase | RPRC014923-PA | 330 | Partial | cd06616 | 24–315 | Yes |
| RpMAP2K6 | *Rhodnius prolixus* | VectorBase | RPRC014787-PA+RPRC014789-PA | 333 | Partial | cd06617 | 45–327 | Yes |
| RpMAP2K7 | *Rhodnius prolixus* | VectorBase | RPRC013126-PA | 372 | Partial | cd06618 | 31–323 | Yes |
| Rpp38 | *Rhodnius prolixus* | VectorBase | RPRC003276-PA | 358 | Complete | cd07851 | 4–346 | Yes |
| RpJNK | *Rhodnius prolixus* | VectorBase | RPRC007591-PA | 389 | Complete | cd07850 | 22–358 | Yes |
| RpERK | *Rhodnius prolixus* | VectorBase | RPRC015063-PA | 362 | Complete | cd07849 | 20–352 | Yes |
| RpMAPK15 | *Rhodnius prolixus* | VectorBase | RPRC008551-PA | 443 | Complete | cl21453 | 21–340 | Yes |

^*^The sequence information of *Homo sapiens*, *Anopheles gambiae*, *Drosophila melanogaster* and *Caenorhabditis elegans* was derived from a previous study (70).

^†^Gene ID represents the gene accession number of the GenBank database (http://www.ncbi.nlm.nih.gov/), Manduca Base (http://agripestbase.org/manduca/), MonarchBase (http://monarchbase.umassmed.edu/) or Vectorbase (http://www.vectorbase.org/).

^‡^The sequences with complete kinase domain were used in the phylogenetic analysis.
