# Supplementary material for: The regulation landscape of MAPK signaling cascade for thwarting Bacillus thuringiensis infection in an insect host
Source: PLoS Pathog. 2021 Sep 8;17(9):e1009917. doi: 10.1371/journal.ppat.1009917 (PMC8452011; doi:10.1371/journal.ppat.1009917)
Supplement: S3 Table — (DOCX) [file ppat.1009917.s012.docx]

**S3 Table. Primers used in this study.**

| Purpose | Genes | Primers | Primer sequence (5′-3′) | PCR product size (bp) | Positions (bp) | TM (°C) |
| --- | --- | --- | --- | --- | --- | --- |
| Full-length cDNA cloning | MAP4K3 | f4K3-F | GCTCGGACATATCCAGAAG | 3444 | 26–3469 | 54 |
|  |  | f4K3-R | CGACTTTGAACTGTAACCCT |  |  |  |
|  | MAP3K4 | f3K4-F | GCGTGTGATAATTTACAATAGTT | 4327 | -26–4301 | 55 |
|  |  | f3K4-R | AGTGTTAAAGTTCACATCTTAGTCT |  |  |  |
|  | MAP3K7 | f3K7-F | CCAAACAATACTGAGAAAATCG | 2297 | -128–2169 | 55 |
|  |  | f3K7-R | ACATCACTCCGGCCTACTT |  |  |  |
|  | MAP3K10 | f3K10-F | GTGTCAACATTTAGTGGTCCGT | 3182 | -203–2979 | 55 |
|  |  | f3K10-R | TTTGAGCCTTCGCTACGAG |  |  |  |
|  | MAP3K12 | f3K12-F | GCGTCTGCTCGTTTCCTC | 1914 | -143–1771 | 55 |
|  |  | f3K12-R | ATCTAAATGTGTGTACAGCCAAA |  |  |  |
|  | MAP3K15 | f3K15-F | ATACTACGCAAATGTTGTGATC | 4236 | -70–4116 | 55 |
|  |  | f3K15-R | CCCGGTAATTGTCAGAGG |  |  |  |
|  | Raf | fRaf-F | TTGCTCGTTTGTCACCATT | 2349 | -46–2303 | 59 |
|  |  | fRaf-R | TGCTCGCTGTCACTGTCC |  |  |  |
|  | TAO | fTAO-F | ATTTTGAGGCGAAATTTTATAGC | 3267 | -103–3164 | 55 |
|  |  | fTAO-R | GGGCGAATGTATTGATTATCTG |  |  |  |
|  | MAP2K1 | f2K1-F | TCCTCTAAATGCCGTGAC | 1278 | -33–1245 | 55 |
|  |  | f2K1-R | TTTTCGCTGTTGAAAGTATG |  |  |  |
|  | MAP2K4 | f2K4-F | TTAGTACCAAAGAATAGTGAACA | 1314 | -47–1267 | 55 |
|  |  | f2K4-R | CCATACGTAACAGTATTTAACC |  |  |  |
|  | MAP2K6 | f2K6-F | TTTTGGAACGAGTTAGAT | 1292 | -70–1222 | 55 |
|  |  | f2K6-R | TCAGACTATTGGGGAAAT |  |  |  |
|  | MAP2K7 | f2K7-F | ATGTGATGTGAGAAAGCGTA | 2131 | -35–2096 | 55 |
|  |  | f2K7-R | TAGGTTATTAACCTCCTATGGT |  |  |  |
|  | p38 | fp38-F | AAAAGTGCTATTCGTGAGTA | 1143 | -23–1103 | 59 |
|  |  | fp38-R | GTAGAACAGGCGTGGTGC |  |  |  |
|  | JNK | fJNK-F | CCCCACTTCTACACGGTCG | 1193 | 43–1235 | 60 |
|  |  | fJNK-R | CGAGTCTTTACACTGTATTCTGCA |  |  |  |
|  | ERK | fERK-F | TGTGATTTTCGGGTAGATTTG | 1160 | -50–1110 | 59 |
|  |  | fERK-R | GGTCCTGCTGGCTGGTGT |  |  |  |
|  | MAPK15 | fK15-F | GATCGTTCGCATGTTAAG | 1764 | -107–1657 | 55 |
|  |  | fK15-R | ATCATCGAATGCCAGTAC |  |  |  |
| qPCR analysis | MAP4K3 | q4K3-F | TGAGAAGGCAACCCTACCG | 150 | 1386–1535 | 52 |
|  |  | q4K3-R | GCCGCAATGCTTTGGTAA |  |  |  |
|  | MAP4K4 | q4K4-F | CATCAACTGGCTCCGTCTG | 188 | 7–215 | 63 |
|  |  | q4K4-R | TCATCTTCGGTGACATCCATC |  |  |  |
|  | MAP3K4 | q3K4-F | AATACGGTTGCCTGTCCTTCG | 152 | 884–1035 | 55 |
|  |  | q3K4-R | GGCTATGAGCGGGTGTTCG |  |  |  |
|  | MAP3K7 | q3K7-F | CGGCACGGAGTAATACCA | 136 | 1484–1619 | 55 |
|  |  | q3K7-R | TAGGGCGTCACATTTCCA |  |  |  |
|  | MAP3K10 | q3K10-F | ACGCCAGCGAAGACATCAA | 146 | 398–543 | 55 |
|  |  | q3K10-R | CGCCGCATACTCCATCACAA |  |  |  |
|  | MAP3K12 | q3K12-F | GCGACAAGAGCGAGACAGA | 147 | 302–448 | 58 |
|  |  | q3K12-R | TGTGCAGGAAGTCGTAGAGC |  |  |  |
|  | MAP3K15 | q3K15-F | TCTCATAGGCAAGAAGGGTA | 145 | 1206–1350 | 58 |
|  |  | q3K15-R | CCAGTTAGGTGGCTTCAGTT |  |  |  |
|  | Raf | qRaf-F | ACAGTGCGAACCCTGGAC | 126 | 586–711 | 55 |
|  |  | qRaf-R | GCAGTAGAAGCCGTTGAAAA |  |  |  |
|  | TAO | qTAO-F | ACCACCAACACTCGTCTCACTC | 160 | 1160–1319 | 52 |
|  |  | qTAO-R | TCCCGTATCGCGTCCCTA |  |  |  |
|  | MAP2K1 | q2K1-F | GAAGGTGCGGCACAAGTC | 158 | 315–472 | 58 |
|  |  | q2K1-R | CACTGTAGAAGGCTCCGTAGAA |  |  |  |
|  | MAP2K4 | q2K4-F | AGGCCACGAAAGAAACTGG | 122 | 43–164 | 55 |
|  |  | q2K4-R | GCTGAGAACGGCACAAACA |  |  |  |
|  | MAP2K6 | q2K6-F | AACTCAAGCAGGTGGTAA | 125 | 791–915 | 58 |
|  |  | q2K6-R | TGCATCATAGTTGGGTCT |  |  |  |
|  | MAP2K7 | q2K7-F | CTCCTCGCTCACCCATTCG | 122 | 1030–1151 | 55 |
|  |  | q2K7-R | CCCATCCTCGGTACATACGC |  |  |  |
|  | p38 | qp38-F | GGCAAGTTTGCTCATCTGT | 102 | 95–196 | 56 |
|  |  | qp38-R | GGTAGGTCCTCTTAGCGTGA |  |  |  |
|  | JNK | qJNK-F | GCTGATGGACGCCAACCTG | 123 | 348–470 | 62 |
|  |  | qJNK-R | CGATGAATAATCCCAGCCAAAT |  |  |  |
|  | ERK | qERK-F | CGCATCACGGTGGAGGA | 145 | 910–1054 | 55 |
|  |  | qERK-R | CGAAGATGAACTGCTTGAGGGT |  |  |  |
|  | MAPK15 | qK15-F | CAAGTCCTATGCGACCTCC | 127 | 1239–1365 | 55 |
|  |  | qK15-R | GCTGATTTCTGATTTGTATTCCCT |  |  |  |
|  | ALP | qALP-F | GCACACACCATGACCGTAGCAG | 169 | 1207–1375 | 61 |
|  |  | qALP-R | GGCTCTTCGTGACATCG |  |  |  |
|  | APN1 | qAPN1-F | TCACTGAGTCCCATCCCA | 157 | 58–214 | 55 |
|  |  | qAPN1-R | TGCCAGACGGCACATTTT |  |  |  |
|  | APN3a | qAPN3a-F | GGCTACCGTTGGCTACAC | 110 | 2190–2299 | 50 |
|  |  | qAPN3a-R | GCAGACATTCCTCCACTCC |  |  |  |
|  | APN5 | qAPN5-F | GGACGATCAGGCTGTTAA | 160 | 2412–2571 | 55 |
|  |  | qAPN5-R | TTCCCGAATCTGGTTGTG |  |  |  |
|  | APN6 | qAPN6-F | CAGCAGGGACGGATAGAA | 146 | 65–210 | 53 |
|  |  | qAPN6-R | ATCGGTAGCAACGAAGTTAA |  |  |  |
|  | ABCB1 | qABCB1-F | AGCGAAAGGAGATTGATAGG | 157 | 942–1098 | 55 |
|  |  | qABCB1-R | GTAATAAACTGGAAACCGAAC |  |  |  |
|  | ABCC1 | qABCC1-F | GGTGGTGCTCATCTGCTACCTCAT | 165 | 696–860 | 55 |
|  |  | qABCC1-R | ATCCTGACACGCTCATCGGTTTT |  |  |  |
|  | ABCC2 | qABCC2-F | AGTCTTGGCACGCAAACGG | 103 | 2401–2503 | 53 |
|  |  | qABCC2-R | CGAACAGACGCATGAAGGACAT |  |  |  |
|  | ABCC3 | qABCC3-F | TCAACCGCTTCACCAAGGACAT | 111 | 2606–2716 | 55 |
|  |  | qABCC3-R | CGGCGTTCAGCACCAGGAT |  |  |  |
|  | ABCG1 | qABCG1-F | ATCTGGTGTTTCAGGCTTTAGTC | 118 | 545–662 | 60 |
|  |  | qABCG1-R | ATCACGGTGTTCTGGCATTT |  |  |  |
|  | RpL32 | qL32-F | CCAATTTACCGCCCTACC | 120 | — | — |
|  |  | qL32-R | TACCCTGTTGTCAATACCTCT |  |  |  |
| dsRNA synthesis^*^ | MAP4K4 | ds4K4-F | T7-GCCCGAGATACGCAAATACA | 582 | 2739–3274 | 55 |
|  |  | ds4K4-R | T7-CCGAGCCATAGATCACTTTCA |  |  |  |
|  | MAP3K7 | ds3K7-F | T7-ACACTGACGCCACGGACAT | 336 | 938–1273 | 55 |
|  |  | ds3K7-R | T7-CAGGCACTGGTGAGCTAGACG |  |  |  |
|  | Raf | dsRaf-F | T7-GAGCAGAGGGAGCGAAGTT | 441 | 271–711 | 55 |
|  |  | dsRaf-R | T7-GCAGTAGAAGCCGTTGAAAA |  |  |  |
|  | TAO | dsTAO-F | T7-ACCACCAACACTCGTCTCACTC | 441 | 1160–1600 | 55 |
|  |  | dsTAO-R | T7-GCTTGTAGCCCGACATCTGC |  |  |  |
|  | MAP2K1 | ds2K1-F | T7-GCGGAGGAGGATAGAAGTGT | 414 | 204–617 | 55 |
|  |  | ds2K1-R | T7-TGCTTGTCCCGCAGGTAG |  |  |  |
|  | MAP2K4 | ds2K4-F | T7-TTCCAAAGCCAAGAACCTC | 342 | 179–520 | 55 |
|  |  | ds2K4-R | T7-CGTAGAAGCGCACAATGTAG |  |  |  |
|  | MAP2K6 | ds2K6-F | T7-TGCCTTACACTACCTATACTCA | 327 | 486–812 | 55 |
|  |  | ds2K6-R | T7-TCTGTTACCACCTGCTTG |  |  |  |
|  | MAP2K7 | ds2K7-F | T7-AACTCCTCGCTCACCCATTC | 480 | 1028–1507 | 55 |
|  |  | ds2K7-R | T7-GTCTCGTCACCTGCTGCTTCT |  |  |  |
|  | p38 | dsp38-F | T7-AAGTGCTATTCGTGAGTAATT | 301 | -21–280 | 55 |
|  |  | dsp38-R | T7-CTTCCAGACACTTCTCCG |  |  |  |
|  | JNK | dsJNK-F | T7-CCGACCACATCGACCAGTG | 311 | 704-1014 | 55 |
|  |  | dsJNK-R | T7-AACCTCCTGCTCGTCATACCA |  |  |  |
|  | ERK | dsERK-F | T7-TTCGGGTAGATTTGTAGAGTTCTG | 350 | -43–307 | 55 |
|  |  | dsERK-R | T7-CCTTCATTTGGTCGATTGAGT |  |  |  |
|  | EGFP | dsEGFP-F | T7-CCACAAGTTCAGCGTGTCCG | 469 | — | 55 |
|  |  | dsEGFP-R | T7-AAGTTCACCTTGATGCCGTTC |  |  |  |

^*^Forward and reverse primers to synthesize specific dsRNA template have the T7 RNA polymerase promoter sequence (5´-TAATACGACTCACTATAGGGAGA-3´) appended to their 5′- and 3′-ends, respectively.
