# Supplementary material for: The regulation landscape of MAPK signaling cascade for thwarting Bacillus thuringiensis infection in an insect host
Source: PLoS Pathog. 2021 Sep 8;17(9):e1009917. doi: 10.1371/journal.ppat.1009917 (PMC8452011; doi:10.1371/journal.ppat.1009917)
Supplement: S4 Table — (DOCX) [file ppat.1009917.s013.docx]

**S4 Table. Primary antibodies used in this study.**

| Protein name | Product number | Manufacturer | Description | Dilution |
| --- | --- | --- | --- | --- |
| p38 | ab170099 | Abcam | Rabbit monoclonal antibody | 1:7500 |
| JNK | ab179461 | Abcam | Rabbit monoclonal antibody | 1:5000 |
| ERK | ab184699 | Abcam | Rabbit monoclonal antibody | 1:2000 |
| p-p38 | CST9215 | Cell Signaling Technology | Rabbit monoclonal antibody | 1:1250 |
| p-JNK | ab4821 | Abcam | Rabbit polyclonal antibody | 1:5000 |
| p-ERK | CST4370 | Cell Signaling Technology | Rabbit monoclonal antibody | 1:2000 |
| β-actin | ab8227 | Abcam | Rabbit polyclonal antibody | 1:2000 |
