# Supplementary material for: The regulation landscape of MAPK signaling cascade for thwarting Bacillus thuringiensis infection in an insect host
Source: PLoS Pathog. 2021 Sep 8;17(9):e1009917. doi: 10.1371/journal.ppat.1009917 (PMC8452011; doi:10.1371/journal.ppat.1009917)
Supplement: Attachment — Submitted filename: Response to Reviewers.docx [file ppat.1009917.s016.docx]

**Response to Reviewers’ comments**

Rachel M McLoughlin, Associate Editor

Michael Otto, Section Editor

Kasturi Haldar, Editor-in-Chief

Michael Malim, Editor-in-Chief

*PLoS Pathogens*

July 29, 2021

Dear Editors,

We would like to thank you, and the three anonymous expert reviewers, for the time spent reviewing our manuscript (Manuscript ID: PPATHOGENS-D-21-01167) entitled "The regulation landscape of MAPK signaling cascade for thwarting *Bacillus thuringiensis* infection in an insect host " submitted to *PLoS Pathogens*. The insightful and constructive comments from the three reviewers have been instrumental in improving the manuscript. We have incorporated their suggested changes in the revised manuscript and highlighted them in yellow. We hope you will find the new version of the manuscript suitable for publication.

The following is a point-by-point response to the reviewer’s comments:

Responses to Reviewers’ Questions

**Part I – Summary**

**Reviewer #1:** In Gou et al., the authors characterize the MAPK signaling cascades that lead to resistance to the Bacillus thuringiensis (Bt) toxin Cry1Ac in the non-model lepidopteran pest Plutella xylostella, the first one for which Bt resistance was reported in the field. They achieved this by mining available genomic and transcriptomic datasets to identify 17 genes encoding for MAPK cascade members in P. xylostella, many of which they found to be overexpressed in P. xylostella strains resistant to Cry1Ac compared to a susceptible strain. The authors also performed quantitative phosphoproteomic profiling of differentially phosphorylated proteins between the midguts of the Cry1Ac resistant P. xylostella strain MIL-R and the Cry1Ac susceptible P. xylostella strain DBM1Ac-S. They found that the phosphorylated versions of the MAPK cascade members MAP3K7, ERK, TAO, MAP2K6 and p38 were significantly more abundant in the resistant strain, compared to the susceptible strain. To elucidate the topology of the MAPK signaling cascades in P. xylostella, the authors used RNAi of 11 MAPK genes and assessed both the phosphorylation of the downstream MAP kinases p38, JNK and ERK by western blot and the susceptibility of larvae to Cry1Ac exposure. These results allowed the authors to reconstruct the topology of the MAPK cascades involved in P. xylostella resistance to Cry1Ac. The authors also showed that treatment with MAPK inhibitors leads to increase in susceptibility to Cry1Ac.

The authors provide exciting new data about the topology of MAPK signaling cascades that contribute to Cry1Ac resistance in the non-model pest P. xylostella. This is novel for this species, however the topology of MAPK signaling is well described in mammals, Drosophila and C. elegans and these known topologies are like what the authors report for P. xylostella. They also show that MAPK inhibitors can restore susceptibility to Cry1Ac in resistant strains, however they don’t provide further evidence of how this could be used in the field to combat pest resistance to Bt. In general, the study presents novel data and an excellent execution and can be recommended for publication in PLoS Pathogens.

We thank reviewer-1 for recognizing the relevance of our data and their positive comments, and we hope that our responses and actions described below will address the raised concerns. Although our last sentence of the manuscript is rather generic in mentioning the potential for using our results for future use in combatting resistant pests, we have given more specific examples (lines 316-320, lines 326-327).

**Reviewer #2:** The authors made an exhaustive study on the MAPK signaling pathways which, in turn, it was previously known that their regulation was altered in some Bt resistant strains. The study shows which pathways are more important determining the susceptibility of P. xylostella to Bt proteins.

We also thank reviewer-2 for recognizing the relevance of the presented data and for their positive comments.

**Reviewer #3:** In this article, the authors conducted a genome-wide identification and characterization of the MAPK signaling cascades and established an MAPK “road map” in the diamondback moth Plutella xylostella. They identified a total of 17 MAPK orthologs in P. xylostella and cloned these MAPK genes. They showed that the transcription levels of most select MAPK cascade genes were up-regulated in the midgut of all resistant P. xylostella strains. They also showed that the phosphorylated protein levels of p38, JNK and ERK were increased in the resistant strains, and that MAP2K6 was involved in activation of p38, MAP2K1 in activation of ERK, and both MAP2K4 and MAP2K7 in activation of JNK. Their data also confirmed that MAP4K4 is the key kinase in activation of all three MAPKs. These results were confirmed by bioassays through RNAi experiment and specific kinase inhibitor assays. Overall, the authors showed that MAPK signaling pathways are involved in regulation of Cry1Ac toxin receptors (ALPs, APNs and ABC transporters), resulting in toxin resistance in P. xylostella. Since p38 pathway can regulate select toxin receptors (for example, APN1, APN3a, APN5, ABCC1, ABCC2 and ABCC3) but not some other receptors (APN6 and ABCB1), and similarly, ERK pathway can regulate select toxin receptors but not some other receptors, it would be great if the authors can elaborate on the similarity and difference of the transcription factor binding sites in the promoter regions of these receptor genes.

We thank reviewer-3 for recognizing the relevance of our data and for their positive comments. In the responses and actions described below, we have attempted to address all of the reviewer’s concerns. Regarding the last comment, we have revised the corresponding section in the Discussion (lines 290-297), where we have discussed the potential role of different TFs.

**Part III – Minor Issues: Editorial and Data Presentation Modifications**

**Reviewer #1: Major Comments**

1. On page 5 of 36 lines 97-99 the authors write: “The fact that resistance in P. xylostella has been linked with multiple and different mechanisms has motivated us to further gain a clear understanding of the underlying interactions, especially for the new-found MAPK signaling pathway” -I would not claim that the MAPK is new-found, this is misleading as much is known about MAPK signaling in response to pore-forming toxins in mammals, Drosophila and C. elegans (which should be mentioned in the introduction to give the reader a broad overview of what is known prior to this paper.) The authors should also emphasize more that MAPK signaling pathways had already been identified to be important in the response against Cry toxins in insects (referencing e.g. previous publications by the authors, https://doi.org/10.1038/srep43964, etc…), but here they explore the full repertoire and function for this particular species (P. xylostella).

We have revised the corresponding section in the Introduction section (lines 99-100, lines 102-107) as suggested by the reviewer.

2. Figure 5 contains some small unlabeled spheres in different tiers of the MAPK signaling pathways, that suggests that the authors suspect that there are additional MAPK cascade genes that they did not identify. The authors should discuss whether this is the case, why their methodology did not pick up these additional members and what could be done in the future to identify them.

The small spheres in the Figure 5 were meant to represent the fact that other MAPK cascade genes exist which may or may not have any role to play in this response. We agree that this is confusing for the reader and so we have deleted them.

3. Using quantitative phosphoproteomic profiling, the authors found that the TAO MAP3K was significantly more phosphorylated in the resistant strain compared to the susceptible strain, however their genetic analyses revealed that TAO had no role in the resistance of P. xylostella to Cry1Ac. Could the authors discuss in more detail why this could have been and also why they didn’t find more MAPK cascade members significantly more phosphorylated in the resistant strain compared to the susceptible strain?

In our quantitative phosphoproteomic analysis, we found that MAP3K7, TAO, MAP2K6, p38 and ERK showed increased phosphorylation in the resistant strain compared to the susceptible strain. We also identified that the phosphorylation level of MAP4K3, MAP3K4, MAP3K12, MAP3K15 and MAP2K7 did not change between these two strains (and have explicitly added this latter observation to the manuscript – lines 177-179). The fact that other MAPK cascades members were not identified in the phosphoproteomic screen may be due to technical limitations in the quantitative phosphoproteomic analysis. Although TAO was found to have increased phosphorylation in the resistant strain, the data in Fig 3D/E/F show no association with resistance. The phosphoprotemic screen was a useful tool to identify possible cascade proteins associated with resistance, which could then be followed up with the RNAi studies. Having failed to find any direct association between TAO expression/phosphorylation and resistance, we do not feel it helpful to speculate on its altered phosphorylation level in the resistant strain. In the manuscript, we have added a sentence (lines 312-314) stating that the role of TAO phosphorylation, if any, is unknown although could be the result of a separate MAP4K4 pathway.

Minor Comments

1. In Fig 1A the authors could include the data from Homo sapiens and C. elegans that they include in Table S2 for reference.

We have revised Fig 1A and the legend of this figure following the reviewer’s suggestion.

2. On page 6 of 36 line 120, the authors claim that the Ka/Ks values calculated for MAPK members reflects low selection pressure. I would disagree with this as values between 0 and 0.25 imply a clear trend towards purifying or stabilizing selection, or selection pressure acting against change in these sequences.

We have revised the corresponding section following the reviewer’s suggestion (lines 126-127).

3. In figure 1C the authors should indicate which branches correspond to MAP3K.

The figure legend states that Raf, MEKK and TAO represent MAP3K, to make it more clear, we have modified the figure adding this information.

4. In Fig S5C there is not so much difference in expression of MAPK cascade genes in fungal infection compared to control. The authors could represent this as Log2Fold Change expression relative to control to highlight this.

The data of Fig S5C used the log2 TPM values of genes which were obtained from RNA-seq. It is clear that the expression of most MAPK cascade genes did not significantly change. We have modified the figure legend to make it clear to the readers that all data analyzed represent Log2Fold change expression levels.

5. Is there no data of P. xylostella exposure to Cry1Ac to include in Fig S5C? This would be more relevant for the purpose of the paper.

The data of Fig S5C were obtained just from a study of fungal pathogen infection (as mentioned in the figure legend), these are not our own data and don’t include Cry1Ac.

6. On page 7 of 36 line 146, the authors should write “(…) genes were up-regulated in the midgut tissues of all resistant strains compared to the susceptible strain DBM1Ac-S.” This enhances clarity for the reader.

We have revised the corresponding sections following the reviewer’s suggestion (line 152-154).

7. The legend of figure 1 should clarify that panels D-F quantify basal expression of MAPK cascade genes without Cry1Ac exposure.

We have revised the figure legend following the reviewer’s suggestion.

8. The legend of figure 2 should clarify that it presents basal differentially phosphorylated proteins between resistant and susceptible strains without Cry1Ac exposure. Also, it should mention that the protein-protein interaction data comes from the STRING database.

We have revised the figure legend following the reviewer’s suggestion.

9. In S8 Fig, at what time were the samples collected to determine the MAPK inhibitor concentration?

The samples were collected at 6 h after treatment to determine the MAPK inhibitor concentration. We have added this information in the S8 Fig legend following the reviewer’s suggestion.

10. The legend of Figure 5 should include the citation of the paper that reports that insect hormones 20E and JH can activate MAPK signaling, since this was not described in the current work.

Revised accordingly.

**Part II – Major Issues: Key Experiments Required for Acceptance**

Reviewer #2: The final conclusion of the study is that the MAPK signaling pathways play an important role in the susceptibility of P. xylostella to Bt proteins. The authors show upregulation and downregulation of some of the genes in the resistant strains. However, it is not clear how this regulation would interfere with the toxic action of the Cry proteins from Bt. In other words, would the increase in the final gene products (membrane receptors) confer resistance to Bt? Or is the depletion of such gene products what would confer resistance? This aspect is not clear in the final conclusions. I think the authors should discuss on this.

In our previous studies (references 30-34 in the main text), we have already performed a lot of studies on ALP, ABC and APN genes. The results indicated that proteins which are encoded by ALP, ABCC2, ABCC3, ABCG1, ABCB1, APN1, APN3a genes function as Bt Cry1Ac receptors. Down-regulation of these genes in the resistant *P. xylostella* strain leads to reduction of Cry1Ac toxin binding to the midgut tissue, which plays an important step in the mechanism of action resulting in higher resistance to Cry1Ac toxin and survival of the larvae. We have added a comment in the Discussion (lines 282-285). Also, see result section (lines 223-231) where we make some relevant comments.

Figure Files:

While revising your submission, please upload your figure files to the Preflight Analysis and Conversion Engine (PACE) digital diagnostic tool, https://pacev2.apexcovantage.com. PACE helps ensure that figures meet PLOS requirements. To use PACE, you must first register as a user. Then, login and navigate to the UPLOAD tab, where you will find detailed instructions on how to use the tool. If you encounter any issues or have any questions when using PACE, please email us at figures@plos.org.

Considering that all of our main figures are big combined figures, we strongly hope they can be published as vector figures, thus, we will submit EPS figures generated by the Adobe Illustrator CC 2015. Moreover, all the supplementary figures will be uploaded as high-quality PDF files.

Data Requirements:

Please note that, as a condition of publication, PLOS' data policy requires that you make available all data used to draw the conclusions outlined in your manuscript. Data must be deposited in an appropriate repository, included within the body of the manuscript, or uploaded as supporting information. This includes all numerical values that were used to generate graphs, histograms etc.. For an example see here: http://www.plosbiology.org/article/info%3Adoi%2F10.1371%2Fjournal.pbio.1001908#s5.

We have added S6 Table that including all the raw data as the supporting information, and revised the corresponding sections in the manuscript (lines 552-553, line 818).

Reproducibility:

To enhance the reproducibility of your results, we recommend that you deposit your laboratory protocols in protocols.io, where a protocol can be assigned its own identifier (DOI) such that it can be cited independently in the future. Additionally, PLOS ONE offers an option to publish peer-reviewed clinical study protocols. Read more information on sharing protocols at https://plos.org/protocols?utm_medium=editorial-email&utm_source=authorletters&utm_campaign=protocols

We have already included the detailed experiment protocols in the manuscript to enhance the reproducibility of our results.

References:

Please review your reference list to ensure that it is complete and correct. If you have cited papers that have been retracted, please include the rationale for doing so in the manuscript text, or remove these references and replace them with relevant current references. Any changes to the reference list should be mentioned in the rebuttal letter that accompanies your revised manuscript. If you need to cite a retracted article, indicate the article’s retracted status in the References list and also include a citation and full reference for the retraction notice.

We confirmed the reference list is complete and correct, and we have added several references as the reviewers’ suggestions.

We have carefully revised the manuscript according to the reviewers’ suggestions. Hopefully, our efforts and the revised manuscript can ease reviewers’ concerns and meet the journal standards. Please contact me if further modifications are required.

Sincerely Yours

Youjun Zhang
